# The effects of rotating magnetic field and antiseptic on in vitro pathogenic biofilm and its milieu

**DOI:** 10.1038/s41598-022-12840-y

**Published:** 2022-05-25

**Authors:** Daria Ciecholewska-Juśko, Anna Żywicka, Adam Junka, Marta Woroszyło, Marcin Wardach, Grzegorz Chodaczek, Patrycja Szymczyk-Ziółkowska, Paweł Migdał, Karol Fijałkowski

**Affiliations:** 1 Department of Microbiology and Biotechnology, Faculty of Biotechnology and Animal Husbandry, West Pomeranian University of Technology, Szczecin, Piastów 45, 70-311 Szczecin, Poland; 2 Department of Pharmaceutical Microbiology and Parasitology, Faculty of Pharmacy, Medical University of Wroclaw, Borowska 211a, 50-534 Wrocław, Poland; 3 Faculty of Electrical Engineering, West Pomeranian University of Technology, Szczecin, Sikorskiego 37, 70-313 Szczecin, Poland; 4 Laboratory of Confocal Microscopy, Łukasiewicz Research Network-PORT Polish Center for Technology Development, Stabłowicka 147, 54-066 Wrocław, Poland; 5 Centre for Advanced Manufacturing Technologies (CAMT/FPC), Faculty of Mechanical Engineering, Wroclaw University of Science and Technology, Łukasiewicza 5, 50-371 Wrocław, Poland; 6 Department of Environment, Hygiene and Animal Welfare, Faculty of Biology and Animal Science, Wroclaw University of Environmental and Life Sciences, Chełmońskiego 38C, 51-630 Wrocław, Poland

**Keywords:** Antimicrobials, Biofilms

## Abstract

The application of various magnetic fields for boosting the efficacy of different antimicrobial molecules or in the character of a self-reliant antimicrobial agent is considered a promising approach to eradicating bacterial biofilm-related infections. The purpose of this study was to analyze the phenomenon of increased activity of octenidine dihydrochloride-based antiseptic (OCT) against *Staphylococcus aureus* and *Pseudomonas aeruginosa* biofilms in the presence of the rotating magnetic field (RMF) of two frequencies, 5 and 50 Hz, in the in vitro model consisting of stacked agar discs, placed in increasing distance from the source of the antiseptic solution. The biofilm-forming cells' viability and morphology as well as biofilm matrix structure and composition were analyzed. Also, octenidine dihydrochloride permeability through biofilm and porous agar obstacles was determined for the RMF-exposed versus unexposed settings. The exposure to RMF or OCT apart did not lead to biofilm destruction, contrary to the setting in which these two agents were used together. The performed analyses revealed the effect of RMF not only on biofilms (weakening of cell wall/membranes, disturbed morphology of cells, altered biofilm matrix porosity, and composition) but also on its milieu (altered penetrability of octenidine dihydrochloride through biofilm/agar obstacles). Our results suggest that the combination of RMF and OCT can be particularly promising in eradicating biofilms located in such areas as wound pockets, where physical obstacles limit antiseptic activity.

## Introduction

The ability to form biofilm is considered one of the most pivotal virulence factors enabling microorganisms adaptation to the environment of the infection site^1^. The biofilm is a highly organized, microbial community containing not only metabolically active but also slow-growing and dormant cells. This multi-cellular society of aggregated microorganisms is enclosed within a self-produced extracellular matrix (ECM). The matrix may account for even 90% of biofilm’s dry mass and may consist of proteins, glycoproteins, polysaccharides, and extracellular DNA^1,2^ in various proportions. The ECM provides bacteria protection, nutrition source, and the environment in which virulence factors or messenger molecules are interchanged rapidly and effectively. Moreover, bacteria distributed in various layers of ECM display metabolic differentiation. This phenomenon is considered an important component of observed high tolerance (reaching up to 1000 times) of biofilm against various antimicrobial agents compared to planktonic (non-adhered) cells of the same microbial strain^3^.

The application of antiseptics (instead of antibiotics) has become successively common in the therapy and prophylaxis of topical, biofilm-related infections, especially these of skin and wounds^4^. The main rationale standing behind it, is antiseptics’ mechanism of action, significantly decreasing the risk of resistant strains emergence. As an example, the molecular activity of octenidine dihydrochloride leads to the destruction of cellular walls and membranes, followed by cytoplasmic leakage, enzymatic malfunctions, and cell death, finally.

Although modern antiseptics are considered efficient antimicrobials, biofilm communities within chronic wounds are often able to survive the treatment and rebuild their structure within a relatively short time^5^. It is due to not only the aforementioned protective properties of biofilm structure but also due to the specificity of the chronic wound environment itself. The majority of chronic wounds produce an exudate, a turbid cellular fluid that dilutes antiseptic concentration, and also binds antiseptic molecules to proteins and/or blood cells (in the case of so-called “fresh bloody exudate”) contained within^6^. Moreover, the topographical irregularities and niches in the wound are used by microbes as a shelter from unfavorable agents (i.e. their active substances cannot reach the site where biofilm develops or they reach there in decreased concentration)^7^. Lastly, such prevalent wound pathogens as *S. aureus*, developed the ability to use host fibrinogen and transform it into fibrin. These fibrin accretions form a physical object referred to as the clot, containing bacteria within it, and protecting them, to a specific extent (depending on the clot size) from antiseptics^8^. Noteworthy, the application of higher concentrations of antiseptics, which would overcome the above-mentioned processes and phenomena, leads to the cytotoxic effect on cells of the wound bed and inhibition of healing^9^. Therefore, the question which should be addressed at this point concerns the possibility of increasing antiseptics’ antibiofilm efficacy without an increase in their concentration. Although such a claim seems to be hard to achieve, the application of various types of magnetic fields intended as an agent boosting the efficacy of different antimicrobial molecules^10–12^ or in the character of a self-reliant antimicrobial agent^13,14^ is considered to be a promising approach.

In our previous works, we have shown that the specific type of magnetic field, referred to as the rotating magnetic field (RMF), acts on the charged molecules (e.g. ions of antimicrobials present in a medium) moving them accordingly to the magnetic field rotation^15^. Furthermore, we previously demonstrated that the RMF displays an impact (of various nature—from negative to positive one) on the growth, metabolic activity, and biofilm formation of several different strains and species of microorganisms^16–18^. In a separate line of investigation, we analyzed the combined effect of RMF and different antibiotics and antiseptics against *S. aureus* and *P. aeruginosa* biofilms in a standard microplate model^19^. The obtained results indicated that the reduction of biofilms exposed to the RMF and antimicrobials was 50% higher as compared to biofilms exposed to antimicrobials only. We have also proved that RMF increases the bactericidal effect of different classes of antibiotics against *S. aureus*, especially methicillin-resistant strains (MRSA)^20^. The observed effect concerned, to the highest extent, these antibiotics, which affect and alter structures of the bacterial cell walls^21^. Although the aforementioned studies were performed not on the bacterial cells within biofilm structure, the obtained results provided us a strong empirical back-up concerning the matter analyzed and moved us to the performance of the present investigation line.

The purpose of this study was to investigate and understand the nature of the increased activity of octenidine dihydrochloride-based antiseptic in the presence of the RMF toward biofilm (including its components, i.e. cells and matrix) and the changes in octenidine dihydrochloride behavior in biofilm milieu (understood as the antiseptic penetrability through the surface the biofilm was cultured on). We hypothesized that the RMF can have an impact on all of the above components. Therefore, we evaluated the changes caused by the RMF (of two distinguished frequencies, 5 and 50 Hz) during the 1, 2, or 3 h exposure on the cell viability and morphology as well as on the structure and composition of biofilms formed by *S. aureus* and *P. aeruginosa* on agar discs, placed in increasing distance from the source of the antiseptic solution at a concentration below the bactericidal effect. Moreover, the penetrability of octenidine dihydrochloride through the porous, agar obstacles as well as the relationship between the RMF parameters and duration of the exposure with regard to the bacterial species (and thus the type of biofilm formed by them) were investigated.

## Materials and methods

### Bacterial strains and antimicrobial

For experimental purposes, the reference American Tissue and Cell Culture (ATCC, USA) strains of *P. aeruginosa* 15442 and *S. aureus* 6538 were used. The applied antimicrobial was an antiseptic containing 0.1% of octenidine dihydrochloride (Schulke-Mayr GmbH, Germany), later referred to as the “OCT”.

### Experimental setup

**Figure 1:**
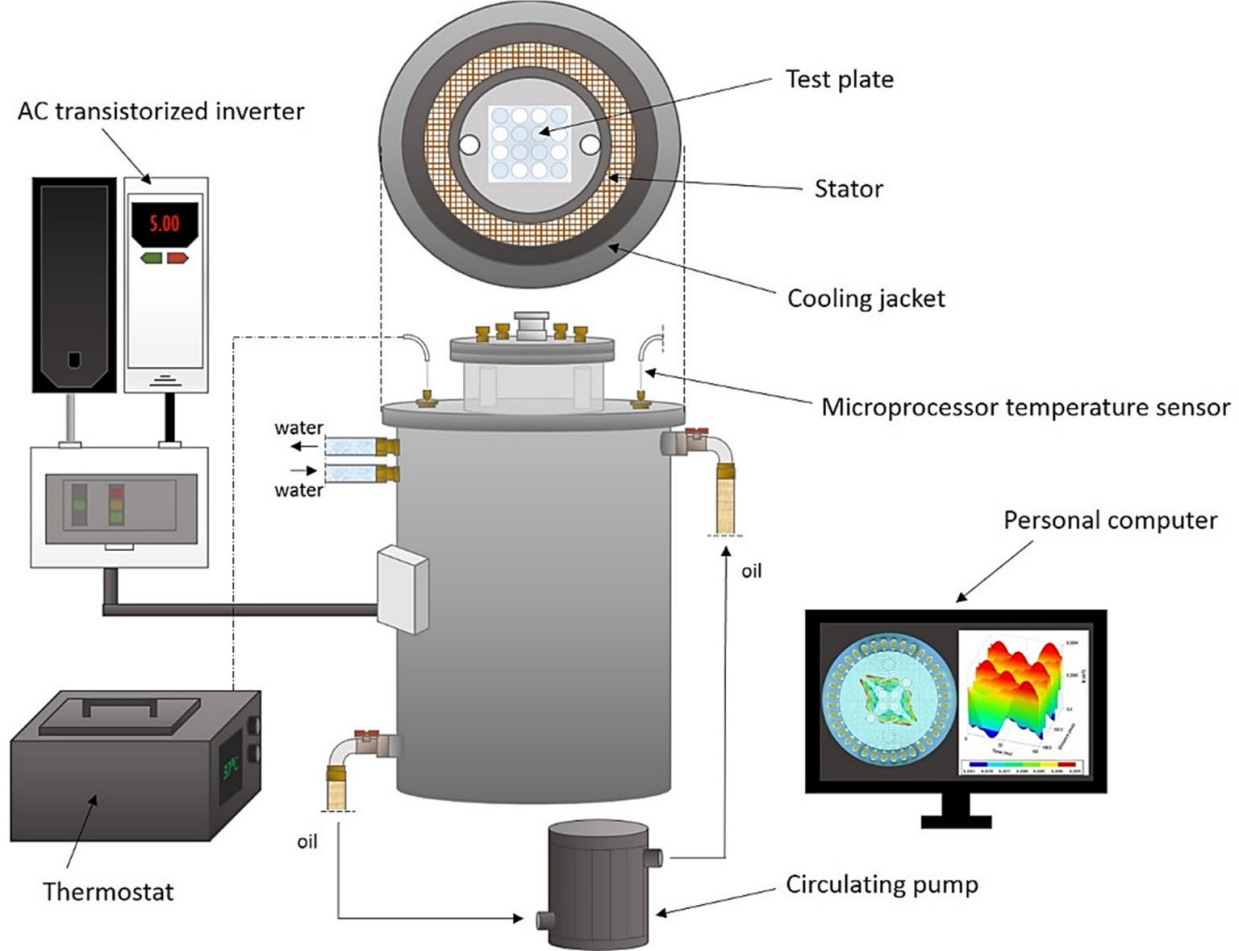
RMF generator with monitoring and control equipment.

The exposure of biofilm to the RMF was carried out using self-designed RMF bioreactors, described in our previous works^18,20,21^, and adopted for purposes of this research. One of the bioreactors was operated with the active RMF generator, while the second served as the control setup (without RMF). Each RMF bioreactor (Fig. 1) was constructed from a 3-phase, four-pole stator consisting of twelve groups of three coil sets. The internal dimensions of the process chamber (in which the incubation of microbiological samples took place) were 16 cm in diameter and 20 cm in height. A detailed description of the RMF generator parameters was provided in Supplementary Table S1. The frequency of alternating current (AC) supplied to the RMF generator was adjusted with the Unidrive M200 inverter (Control Techniques, Nidec Industrial Automation, Poland). The temperature inside the RMF process chamber was regulated and corrected using a thermostat (KISS K6, Huber, Germany) connected to a circulating pump system (Yonos Pico 2.0 25/1-6 25/60, Wilo, Ireland) and the heat exchanger equipped with temperature probes (LM-61B, National Semiconductor Corporation, USA). The homogeneous temperature distribution in the RMF bioreactors was maintained by air flow provided during exposure (1 L/min, 35 °C, RH 90%). The distribution of magnetic induction (*B*) in the process chamber was determined at 100 V and AC frequencies of 5 and 50 Hz using the Ansys Maxwell simulation software ver.19.1 (ANSYS Inc., USA) and measured using teslameter (SMS-102, Asonik, Poland).

### Preparation of OCT containing carrier

The discs made of bacterial cellulose (later referred to as the carriers) synthesized, purified and dried as described in earlier works of our team^22,23^ of 15 mm diameter and 0.015 mm thickness were saturated by immersion with OCT dilutions in PBS (Millipore Sigma, USA), (1:16 for *S. aureus* biofilm and 1:4 for *P. aeruginosa* biofilm) for 24 h at room temperature. Above dilutions were experimentally selected because they did not lead to an antibiofilm effect in 3 h contact time (Supplementary Fig. S1). Additionally, to prepare the control setting for the experiments based on using OCT, the carriers were soaked with PBS instead of the antimicrobial.

Based on the difference in weight (measured using an analytical balance, Radwag, AS 160.R2, Poland) between the dry and the impregnated carrier, it was found that the volume of the OCT solution (or PBS) absorbed by the disc was 25.0 µL.

### Antibiofilm dressing’s activity measurement test

To analyze the antibiofilm activity of the OCT released from the carrier, the Antibiofilm Dressing’s Activity Measurement (A.D.A.M) test was performed according to the protocol originally devised in our laboratory^2,22^ with minor modifications (concerning the concentration of microorganisms and the size of the agar discs). Briefly, *P. aeruginosa* ATCC 15442 and *S. aureus* ATCC 6538 colonies grown on the Columbia Agar (containing 5% sheep blood medium) were transferred into 5 mL of Tryptic Soy Broth (TSB) medium and incubated for 24 h at 37 °C with shaking (200 rpm). After incubation, cultures were diluted in TSB (Biomaxima, Poland) broth supplemented with 1% glucose to obtain the same optical density (OD) equals 1 × 10^3^ CFU/mL. Simultaneously, agar discs of 6 mm in diameter and 4 mm high were cut out from the agar plate containing 2% (v/w) bacteriological agar (Graso Biotech, Poland), transferred to the wells of the 24-well plate, immersed in 2 mL of the bacterial suspension and incubated for 48 h at 37 °C to form a biofilm layer on their surface. After incubation, the discs were rinsed 3 times with 2 mL of 0.9% NaCl to remove non-adherent bacteria and transferred to the agar-filled wells of a trimmed 24-well plate with previously cut holes of diameters equal to the agar discs. Three biofilm-containing discs were placed one into another in each of the holes. The disc put on the bottom of the well are further referred to as the “B-disc” (bottom disc); the disc put directly on the B-disc, as the “M-disc” (middle disc); and the last disc put directly on the M-disc, as the C-disc (contact disc). Next, the carriers impregnated with OCT (or PBS) were placed directly on the C-disc and the plate was covered with a lid.

### Exposure of biofilms to RMF

**Figure 2:**
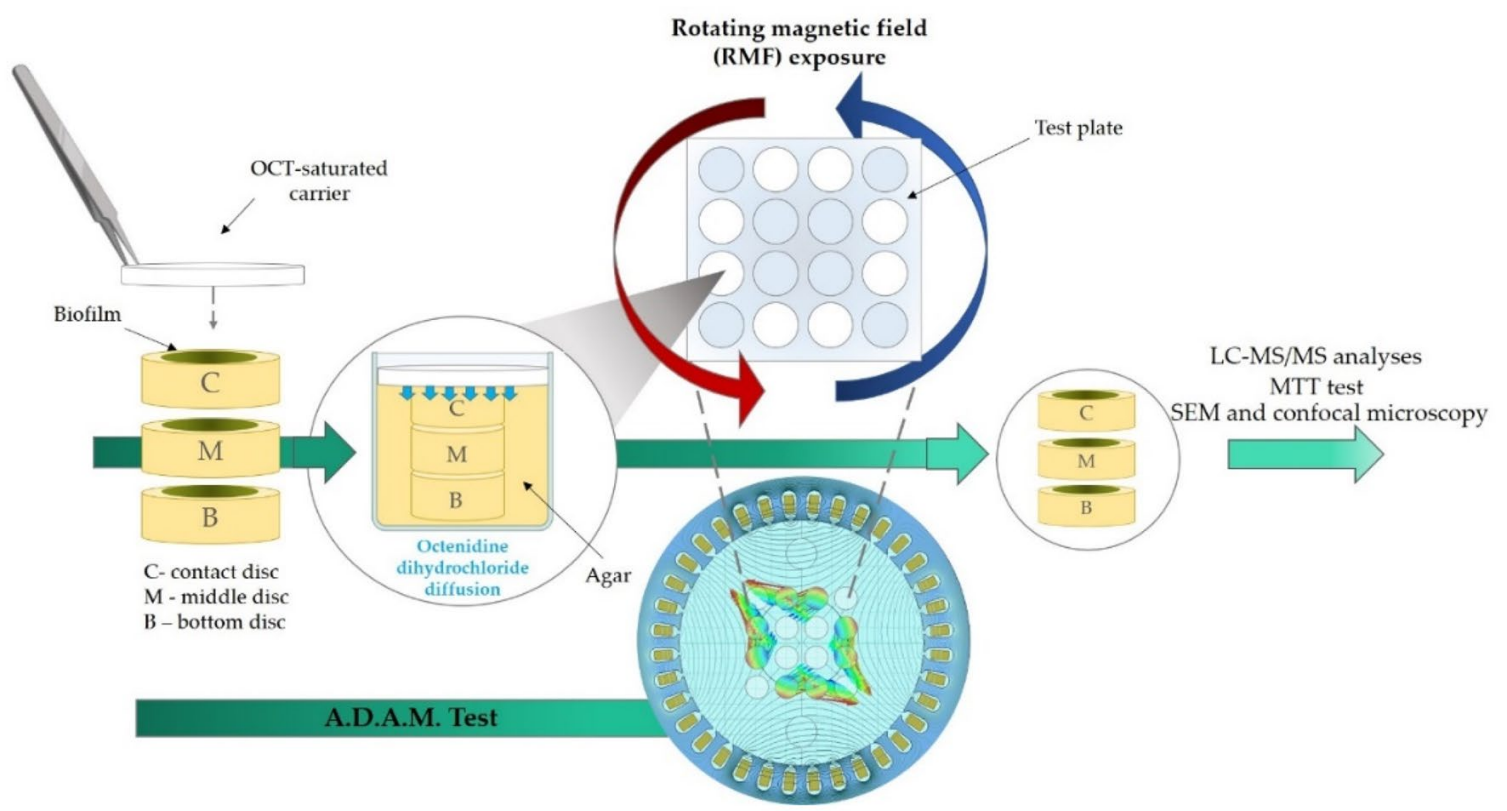
The graphical presentation of the A.D.A.M. test and the arrangement of the test plate during the exposure to rotating magnetic field (RMF).

To ensure uniform exposure to the RMF, each 24-well plate (later referred to as the “test plate”) was aseptically trimmed with two end columns (columns A and F) before the A.D.A.M test setting preparation. In turn, the biofilm-containing discs were placed only in the outer wells, excluding the corner wells. Thanks to such distribution, all biofilm samples exposed to the RMF were at the same distance from the stator. The test plate with biofilm was placed in the center of the RMF generator (thus subjected to the influence of a magnetic field characterized by the same parameters) and in the middle of the height of the RMF generator (where the magnetic induction value was maximal, Supplementary Fig. S2). The graphical presentation of the arrangement of the test plate during the exposure to the RMF is presented in Fig. 2.

The test plates were exposed to the RMF of 5 and 50 Hz (the minimal and maximal frequencies that can be set in the experimental setup) for 1, 2, or 3 h at 37 °C. In the case of exposure lasting for 1 and 2 h, the plates were further incubated until 3 h (for 2 and 1 h, respectively) maintaining the same conditions, however with the RMF generator switched off.

The control RMF-unexposed plates were incubated in the twin bioreactor, which had the RMF generator switched off (Supplementary Fig. S3). This RMF-off bioreactor was arranged approximately 2 m from the RMF-on bioreactor. As measured using a Hall probe (Smart Magnetic Sensor-102, Asonik, Poland), the source of the RMF did not affect the RMF-off bioreactor during the experiment (the magnetic induction (*B*) inside the RMF-off bioreactor was ≤ 0.05 mT).

### Evaluation of bacterial viability in biofilm

After 3 h of RMF exposure and/or incubation, the carriers were removed, agar discs covered with biofilm were carefully transferred to a 24-well plate containing 2 mL of MTT (3-(4,5-dimethylthiazol-2-yl)-2,5-diphenyltetrazolium bromide, Millipore Sigma, USA) solution (3 mg/mL in TSB) and incubated in the dark for 1 h at 37 °C. After incubation, the discs were transferred to 2 mL of isopropanol and shaken vigorously using a plate shaker for 15 min to dissolve the formazan resulting from MTT reduction. After shaking, the MTT-formazan solution was aspired and transferred to the wells of a 96-well plate. Finally, the absorbance of the solution obtained was measured with a wavelength of 570 nm and a reference wavelength of 690 nm using a microplate reader (Tecan, Infinite 200 PRO NanoQuant, Switzerland).

Results were presented as % of living cells on successive agar discs for the experimental setting in comparison to the control setting calculated by the following formula (1):

1 $$\%\, of\, living\, cells =\left(\frac{\left({Abs}_{experimental} -{Abs}_{background}\right)}{\left({Abs}_{control}-{Abs}_{background}\right)}\right)\times 100$$

where *Abs*_*experimental*_ is the absorbance of MTT-formazan solution obtained for biofilms from the experimental settings, *Abs*_*control*_ is the absorbance of MTT-formazan solution obtained for biofilms from the control settings, *Abs*_*background*_ is the absorbance of the experimental or control samples containing no bacteria (biofilm).

Depending on the purpose of the analysis, different comparative experimental and control settings were used: (i) to determine the antibiofilm activity of OCT solution, results were calculated as % of living biofilm-forming cells on successive agar discs treated with OCT-saturated carriers (OCT-treated biofilms) in comparison to the setting with the carriers soaked with PBS instead of OCT (PBS-treated biofilms). In this setting all the biofilm samples were incubated in the control bioreactor (RMF-off generator), (ii) to determine the effect of RMF exposure on the viability of biofilm-forming bacteria, results were calculated as % of living biofilm-forming cells on agar discs exposed to RMF (RMF-exposed biofilms) in comparison to the setting unexposed to RMF (RMF-unexposed biofilms). In this setting all biofilm samples were treated with the carriers soaked with PBS (instead of OCT), (iii) to determine the influence of RMF on antibiofilm activity of OCT solution (main experimental settings), results were calculated as % of living biofilm-forming cells on agar discs treated with OCT-saturated carriers and exposed to RMF (OCT-treated/RMF-exposed biofilms) in comparison to the setting with the carriers soaked with OCT but unexposed to RMF (OCT-treated/RMF-unexposed biofilms).

### Analysis of impact of RMF on penetration rate of octenidine dihydrochloride

In order to analyze the impact of RMF on the penetration rate of octenidine dihydrochloride (the main active component of applied antiseptic product) through successive agar discs, the same A.D.A.M.-based setting as the one used for the measurement of antibiofilm activity was exposed to the RMF generated at 5 and 50 Hz (or incubated in RMF-off condition). However, for this study, the agar discs (C, M, B) were not covered by biofilm and the carrier was impregnated with the original OCT solution (without a dilution). The remaining analysis conditions, including the temperature and humidity as well as exposure and incubation time, were the same as during the measurement of antibiofilm activity.

To extract the octenidine dihydrochloride, the agar discs were placed in 0.5 mL of methanol (Stanlab, Poland) in deionized water (1:1) and incubated with shaking (250 rpm; Biosan, PSU-10i, Latvia) for 3 h^20,21^. Next, the methanol–water mixtures with the extracted antiseptics were filtered through a syringe filter (0.22 µm pore diameter, Chromafil^®^ Xtra, Macherey–Nagel, Germany) and analyzed by liquid chromatography–tandem mass spectrometry (LC–MS/MS) technique (1260 Infinity II Series Liquid Chromatograph, Agilent, USA). An InfinityLab Poroshell 120 EC-C18 column (Agilent, USA) with a particle diameter of 2.7 µm equipped with a guard column was used for chromatographic separation. A mass spectrometer (Ultivo G6465B, Agilent, USA) coupled to the chromatograph was used to detect and identify the assessed antiseptics. The quantitative analysis was based on calibration curves prepared with the use of octenidine dihydrochloride standards (Dishman Pharmaceuticals & Chemicals Ltd., UK). The results were presented as % of octenidine dihydrochloride extracted from successive agar discs exposed to RMF (or incubated in RMF off conditions) in comparison to its initial concentration in the carrier.

### Visualization of cell wall integrity of biofilm-forming cells

In order to visualize the impact of RMF and OCT (together or as self-reliant agents) on microbial biofilm, the biofilm samples were immersed in 1 mL of Filmtracer^™^ LIVE/DEAD^™^ Biofilm Viability Kit (Invitrogen, Thermo Fisher Scientific, USA) solution and incubated at room temperature for 15 min^20,21^. After incubation, the solution was removed and the biofilms were gently rinsed once with sterile water. The biofilms were analyzed using a confocal microscope (Leica, SP8, Germany) with a 25x water dipping objective, using sequential mode for 488 nm laser line and 500–530 nm emission to detect SYTO-9 and 552 nm laser line and 575–627 nm emission to detect propidium iodide (PI) within microbial cells. The subsequent images (biofilm cross-sections) were collected with ~ 2 µm spacing in the Z dimension. The obtained biofilm images were further analyzed with Imaris 9 (Abingdon, UK) software, with the use of the maximum intensity projection method.

The following settings of this experiment were prepared: OCT-treated/RMF-exposed biofilms; OCT-treated/RMF-unexposed biofilms; PBS-treated/RMF-exposed biofilms; PBS-treated/RMF-unexposed biofilms. The analysis conditions, including OCT dilution, temperature and humidity as well as exposure and incubation time, were the same as during the measurement of antibiofilm activity (A.D.A.M. test).

### Visualization of cells, structure of biofilm matrix and porosity of agar discs

To confirm the presence of biofilm formed on agar discs, to visualize the impact of RMF on the biofilm structure and cell status, and to visualize the porosity of agar discs, scanning electron microscopy (SEM) was applied. The PBS-treated/RMF-exposed and PBS-treated/RMF-unexposed biofilms prepared in the same conditions as in the A.D.A.M. test were rinsed with PBS and fixed by immersion in 3% glutarate (POCH, Poland) for 15 min at room temperature. Next, the biofilms were rinsed twice with PBS to remove the fixative. The dehydration in increasing concentrations of ethanol (POCH, Poland) (25%, 50%, 60%, 70%, 80%, 90%, and 100%) was performed for 10 min per solution. Then, the ethanol was rinsed off, and the biofilms were dried at room temperature. Next, biofilms were covered with gold and palladium (60:40; sputter current, 40 mA; sputter time, 50 s) using a Quorum machine (Quorum International, USA) and examined using the SEM (Carl Zeiss, EVO MA25, Germany). In the case of agar discs without biofilm (analysis of agar discs porosity), the procedures were analogical as in the case of biofilm-covered discs, the procedures of fixation and visualization were analogical as in the case of biofilm-covered discs, with the exception that the first step (rinsing with PBS) was omitted.

The SEM images of PBS-treated/RMF-exposed and PBS-treated/RMF-unexposed biofilms and agar surfaces were processed using ImageJ software (National Institutes of Health, USA). The images were converted into 16-bit images and considered Regions of Interest (ROIs). Next, the threshold was settled for ROIs in such a manner that fibrils were recognized as background (“Image/Adjust/Threshold” command), while the pores were recognized as the areas to be further analyzed. The “Process/Subtract” command was applied to manage the background pixels; the number of pores was calculated using the option of “Particles Analyses” with circularity from 0.00 to 1.00. The ferret diameters were calculated using the “Set measurements/Ferret Diameter” command from threshold 16-bit images; each setting was analyzed in six ROIs.

### Determination of saccharide content in cell-free biofilm matrix

Initially, all the strains were plated onto the Columbia Agar (containing 5% sheep blood medium, Graso Biotech, Poland) and cultivated for 24 h at 37 °C. After incubation, one colony-forming unit (CFU) was transferred into 5 mL of Tryptic Soy Broth (TSB, Oxoid, UK) and incubated for another 24 h at 37 °C with shaking (200 rpm, Biosan, ES-20/60, Latvia). Next, cultures were diluted in TSB supplemented with 1% glucose to obtain bacterial suspension equal to 1 × 10^3^ CFU/mL, which was then transferred to Petri dishes (20 mL/Petri dish) and incubated for 48 at 37 °C. After that time biofilms were exposed to the RMF of 5 and 50 Hz (RMF-exposed biofilms) or incubated in RMF-off conditions (RMF-unexposed biofilms) for 3 h. After exposure, the medium was gently removed, and the biofilm, remaining on the dish, was rinsed with 10 mL of PBS. In the next stage, the biofilm was collected using a tissue culture scraper, transferred to a 50 mL test tube, and mixed with 36% aqueous formaldehyde solution (Millipore Sigma, USA). The formaldehyde solution was added in a proportion of 60 µL per 10 mL of biofilm. The resulting suspension was shaken (100 rpm, Biosan, PSU-10i, Latvia) at room temperature for 1 h, then mixed with 1 M NaOH solution (in the proportion of 4 mL of NaOH solution per 10 mL of biofilm suspension), shaken (100 rpm) at room temperature for a further 3 h and centrifuged at 4500 × g for 1 h at 4 °C (Eppendorf, Centrifuge 5804R, Germany). After centrifugation, the supernatants, containing biofilm matrices, were filtered through a syringe filter (0.22 µm pore diameter) and then dialyzed using dialysis membranes (molecular weight cut-off = 14,000, Millipore Sigma, USA) immersed in beakers containing 1 L of DI water. Dialysis was carried out for approx. 3 days at 4 °C (during dialysis, water was changed every 24 h) until the biofilm matrix suspensions reached the resistance of deionized water used for dialysis (resistivity < 1(MWxcm)).

The matrices were hydrolyzed with the use of 80% trifluoroacetic acid (TFA, Milipore Sigma, USA) and evaporated using SpeedVac device SRF110 (Thermo Fisher Scientific, USA). Next, dried samples were derivatized by heating (60 °C for 30 min) in the solution containing 1000 µL of anhydrous pyridine (Millipore Sigma, USA), *N*-Methyl-*N*-tert-butyldimethylsilyltrifluoroacetamide (MTBSTFA, Millipore Sigma, USA), and 100 µL of trimethylchlorosilane (TCMS, Millipore Sigma, USA). The 5 mg of d-sorbitol (Milipore Sigma, USA) was introduced to the solution as an internal standard.

The assessment of saccharide content in the cell-free biofilm matrix was performed using gas chromatography-mass spectrometer coupled with a mass spectrometer single quadrupole (GC–MS/MS, Shimadzu, QP 2010, Japan) with the TRACE^™^ TR-5MS capillary column (0.25 µm, 0.25 mm I.D., 30 m length, Thermo Fischer Scientific, USA). The mobile phase was helium 99.999% (LindeGas, Poland) with a linear velocity of 30 cm/s in constant flow mode. The dosing was set for 1 µL in splitless mode. The inlet temperature was set to 320 °C, ion source temperature was 300 °C. The initial oven temperature was set at 100 °C, increasing 10 °C/min to a final temperature of 320 °C with a 10-min hold time. The mass spectrometer was set to scan mode 10 000 scan/sec in the range 40–600 amu. The spectra were analyzed using GCMSsolution ver. 4.1 software (Shimadzu, Japan) with NIST 14 Library (National Institute of Standards and Technology, USA). All saccharides serving as comparative references were purchased from Milipore Sigma, USA.

#### Confirmation of a lack of cells within the biofilm matrix

To confirm that the purified biofilm matrix contained no cells within, the cryo-SEM technique was applied. The 10 µL of the matrix was placed on the cryo-SEM table. Next, the sample and the table were frozen in liquid nitrogen under vacuum conditions. A vacuum of 10^–7^ mbar was provided in the preparation chamber by a turbomolecular pumping system (Vacuubrand, Germany). The frozen table and the sample were transferred into a vacuum chamber of cryo-SEM attachment (Quorum, UK) and sputtered with a platinum layer. Such prepared sample was examined using SEM (Carl Zeiss, Auriga 60, Germany). The observation was performed at a working distance of 5 mm, EGT = 2 kV/m. The SEM cold stage was − 180 °C with temperature stability of < 0.5 °C.

### Statistical analysis

The data were presented as mean values ± standard error of the mean (SEM) obtained from at least three different measurements (plus technical repetitions). Statistical differences between RMF-exposed and control settings were determined by one-way analysis of variance (ANOVA). Tukey's multiple comparisons test was used for multiple comparisons of means (the post-hoc analysis). Differences were considered significant at a level of *p* < 0.05. The statistical analyses were conducted using GraphPad Prism 9.0 (GraphPad Software Inc., USA).

## Results

### Biofilm formation on agar discs

**Figure 3:**
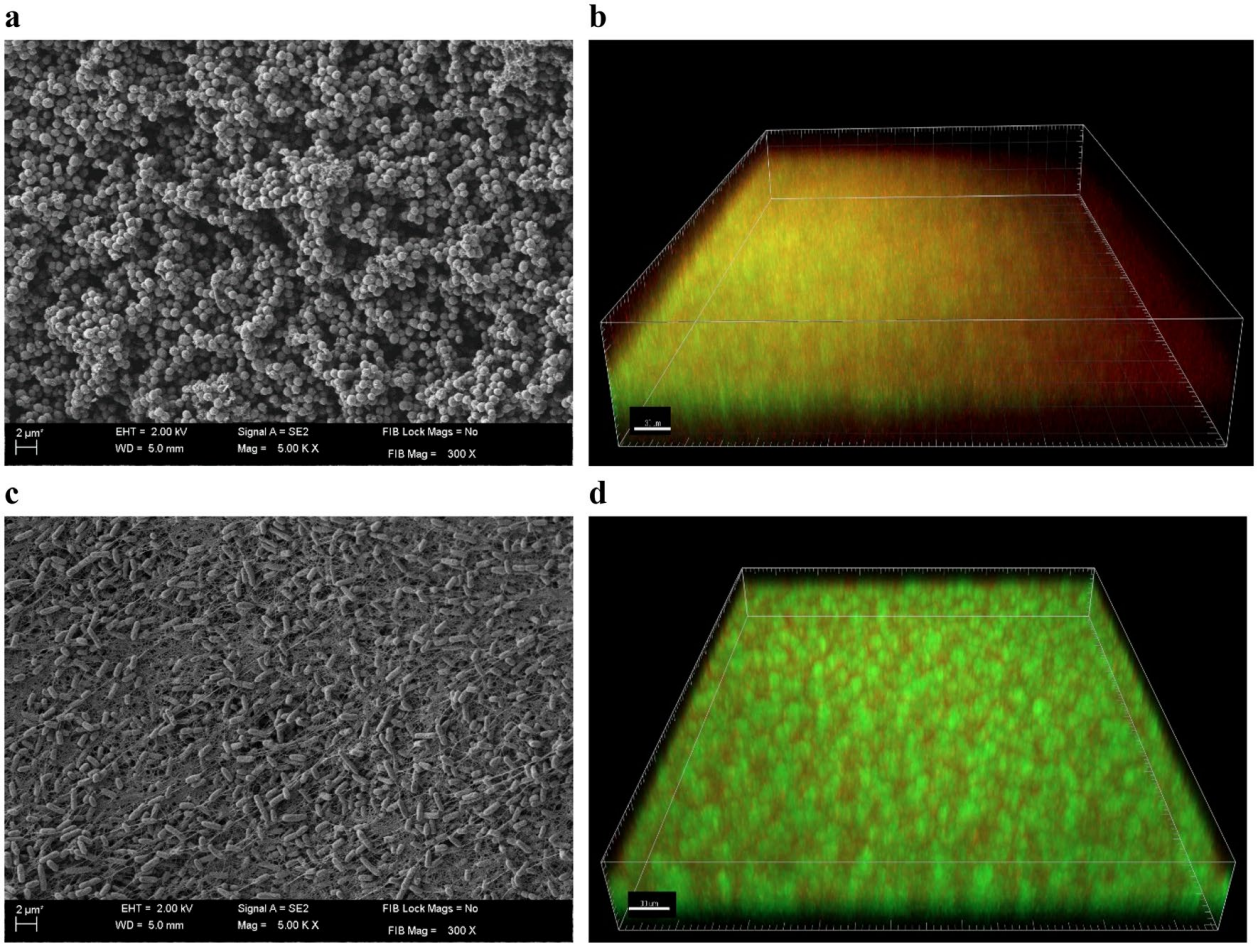
Structure of 48 h biofilm formed on agar discs visualized by scanning electron and confocal microscopy, (**a**, **b**) *S. aureus* and (**c**, **d**) *P. aeruginosa*. The magnification of pictures taken using SEM equals 5000× and confocal microscopy 40×.

Both investigated bacterial species were able to form biofilm structures in the experimental settings applied, as proven through SEM and confocal microscopy (Fig. 3). The observed share of extracellular matrix was higher in the case of *P. aeruginosa* ATCC 14452 than in the case of *S. aureus* ATCC 6538, nevertheless both microbial species formed adhered multi-cellular structures meeting the established criteria of biofilm formation. After proving the ability to produce biofilm in the applied experimental setting, we performed a series of experiments to investigate the impact of RMF combined with the OCT on overall antibiofilm activity, understood as activity against biofilm-forming cells and the ability to penetrate through physical obstacles and biofilm layers formed at different distances from the OCT source. The above analyzes were preceded by the assessment of the influence of OCT (without RMF) and RMF (without OCT) on changes in the viability of biofilm-forming cells.

### Effect of OCT treatment on viability of biofilm-forming bacteria

**Figure 4:**
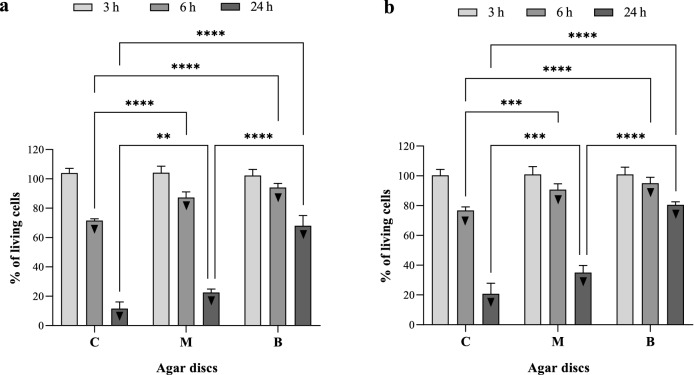
Percent of living (**a**) *S. aureus* and (**b**) *P. aeruginosa* biofilm-forming cells on successive agar discs incubated with OCT-saturated carriers. The results are presented as % reduction of living biofilm-forming cells on successive agar discs incubated with OCT-saturated carriers in comparison to the control setting with the carriers soaked with PBS instead of OCT and expressed as a mean ± SEM. The asterisks in the graphs indicate statistically significant differences between OCT-treated biofilms on successive agar discs (*p < 0.05, **p < 0.01, ***p < 0.001, ****p < 0.0001). ▼—statistically significant differences (*p* < 0.05) between OCT-treated and PBS-treated biofilms.

It was found that during 3 h of contact time, the OCT released from the carrier had no significant impact on the % of viable biofilm-forming cells of both *S. aureus* and *P. aeruginosa* bacterial species, on any of the agar discs (C, M, B). However, the OCT released from the carrier significantly reduced the % of viable biofilm-forming cells when the contact time was exceeded (Fig. 4). In this case, the strongest bactericidal effect was noted for disc C (neighboring with OCT-containing carrier), then for disc M, while the lowest effect was found for disc B (placed in the greatest distance from OCT-containing carrier). The results from the setting applying contact time longer than 3 h (control of testing method usability) confirmed that OCT possesses the appropriate antibiofilm activity, contrary to setting when 3 h contact time was applied. Therefore 3 h of contact time was chosen for analyses of boosting effect of the RMF on OCT activity.

### Effect of RMF exposure on viability of biofilm-forming bacteria

In the next stage of our research, the analysis covered the effect of exposure to the RMF of different frequencies (5 and 50 Hz) lasting 1, 2, and 3 h on changes in the viability of biofilm-forming bacteria on each of the agar discs. It was found that the exposure of biofilm samples to the RMF (PBS-treated and RMF-exposed biofilms) did not significantly affect the viability of *S. aureus* and *P. aeruginosa* cells regardless of the exposure duration and RMF frequencies (5 v. 50 Hz). There were also no statistically significant differences in the viability of RMF-exposed biofilm-forming cells between the individual agar discs (C, M, B), (Supplementary Fig. S4).

### Influence of RMF on OCT antibiofilm activity

**Figure 5:**
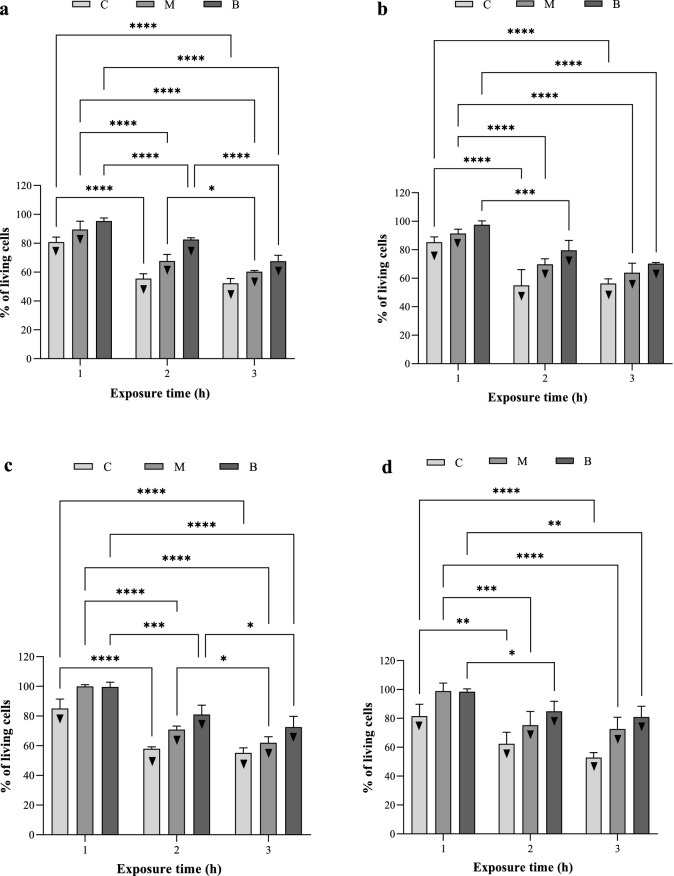
Percent of living biofilm-forming cells on successive agar discs treated with OCT-saturated carriers and exposed to RMF. (**a**) *S. aureus*—RMF of 5 Hz, (**b**) *S. aureus*—RMF of 50 Hz, (**c**) *P. aeruginosa*—RMF of 5 Hz and (**d**) *P. aeruginosa*—RMF of 50 Hz. The results are presented as % reduction of living biofilm-forming cells on successive agar discs treated with OCT-saturated carriers and exposed to RMF in comparison to the settings with the carriers soaked with OCT but unexposed to RMF and expressed as a mean ± SEM. The asterisks in the graphs indicate statistically significant differences between OCT-treated biofilms exposed to RMF for different time (*p < 0.05, **p < 0.01, ***p < 0.001, ****p < 0.0001). ▼—statistically significant differences (*p* < 0.05) between OCT-treated/RMF-exposed and OCT-treated/RMF-unexposed biofilms.

In the further stages of the experiment, it was shown that the exposure of biofilm samples to the RMF (regardless of its frequency) increased significantly the antibacterial effect caused by OCT against *S. aureus* and *P. aeruginosa* biofilms (Fig. 5). The effect was mainly dependent on the time of exposure to the RMF and on biofilm, with regard to the specific agar disc (C, M, B) it grew, and to a lesser extent on the biofilm-forming species or the RMF frequency. Although the observed antibacterial effect was proportional to the exposure time, even the exposure lasting 1 h (the shortest exposure time) caused a significant reduction in the number of viable cells in the *S. aureus* biofilm (formed on discs C and B) and in the case of *P. aeruginosa* biofilm (on the disc C). Nevertheless, the greatest RMF influence was found for the settings exposed for 3 and thereafter for 2 h, where the reduction in biofilm viability (regardless of species and frequency) was statistically significant in all analyzed agar discs (C, M, B). Moreover, the differences between 2 and 3 h exposure to the RMF were of relatively minor level and did not exceed 10%. In the case of a 3 h exposure, the *S. aureus* biofilm viability dropped, respectively, by approx. 45%, 40%, and 30% for the C, M, and B agar discs, and by 45%, 30%, and 20%, during 2 h of exposure. In the case of *P. aeruginosa* biofilm, especially in the 3 h exposure variant, the influence of the RMF frequency was of greater importance for the obtained results. Only the results concerning the biofilm on the C disc were similar for both frequencies (approx. 45%). In turn, in the case of the M and B discs, the differences in viability inhibition, depending on the RMF frequency, were approx. 10% (5 Hz, disc M—40%, disc B—30%; 50 Hz, disc M—30%, disc B—20%). The respective values recorded for 2 h exposure were less substantial and did not exceed 5%—the drop of viability was approx. 40% for the C disc; 25% for the M disc and 17% for the B disc. In the case of exposures lasting 1 h, regardless of the bacterial species and the frequency of RMF, the decrease in cell viability did not exceed 20%. Moreover, in the case of discs M and B, no statistically significant differences were found between the RMF-exposed and RMF-unexposed samples, and the reduction of viability was less than 10%.

Nevertheless, despite the observed differences between individual agar discs depending on the RMF frequency, the total antimicrobial effect of OCT and RMF on C, M, and B discs was comparable, and the differences were not statistically significant, regardless of the analyzed species of bacteria (Supplementary Fig. S5).

### Influence of RMF on release and penetrability rate of octenidine dihydrochloride

**Figure 6:**
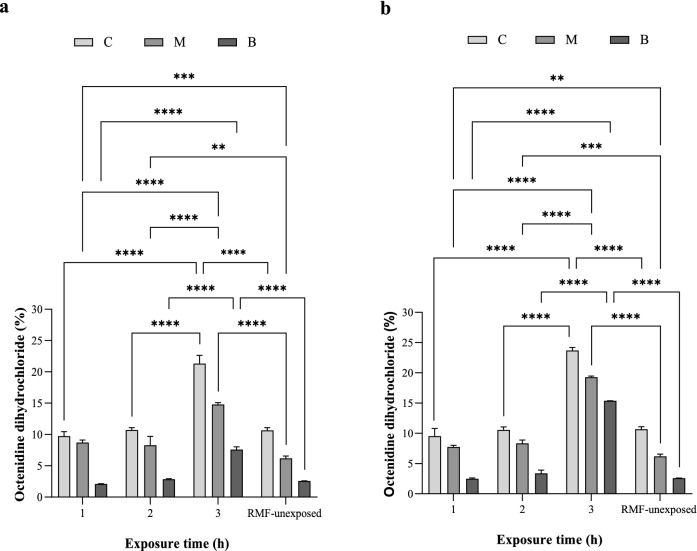
Percent of octenidine dihydrochloride extracted from successive agar discs exposed to RMF of (**a**) 5 Hz and (**b**) 50 Hz, in comparison to its initial concentration in the carrier. The results are presented as mean ± SEM. The asterisks in graphs indicate statistically significant differences between RMF-exposed and RMF-unexposed agar discs (*p < 0.05, **p < 0.01, ***p < 0.001, ****p < 0.0001).

It was found that the RMF altered the level of octenidine dihydrochloride penetration into the individual agar discs (Fig. 6). The most significant influence of the RMF was observed during continuous exposure for 3 h. In this case, the greater effect was observed as a result of exposure to the RMF of 50 Hz (all discs consisted of a total of 58% of octenidine dihydrochloride as compared to its initial concentration in the carrier), compared to exposure to the RMF of 5 Hz (43%) (Supplementary Fig. S6). When exposed to the RMF for a shorter time, the octenidine dihydrochloride concentration was also higher as compared to the unexposed control settings, but the differences did not exceed 3%, and significant differences occurred only for M discs, regardless of the RMF frequency. The greatest concentration of octenidine dihydrochloride was detected in disc C and the lowest in the disc placed at the bottom of the test plate (disc B). This trend was observed regardless of the time of exposure to the RMF.

### Influence of RMF on biofilm-forming cells and biofilm matrix

**Figure 7:**
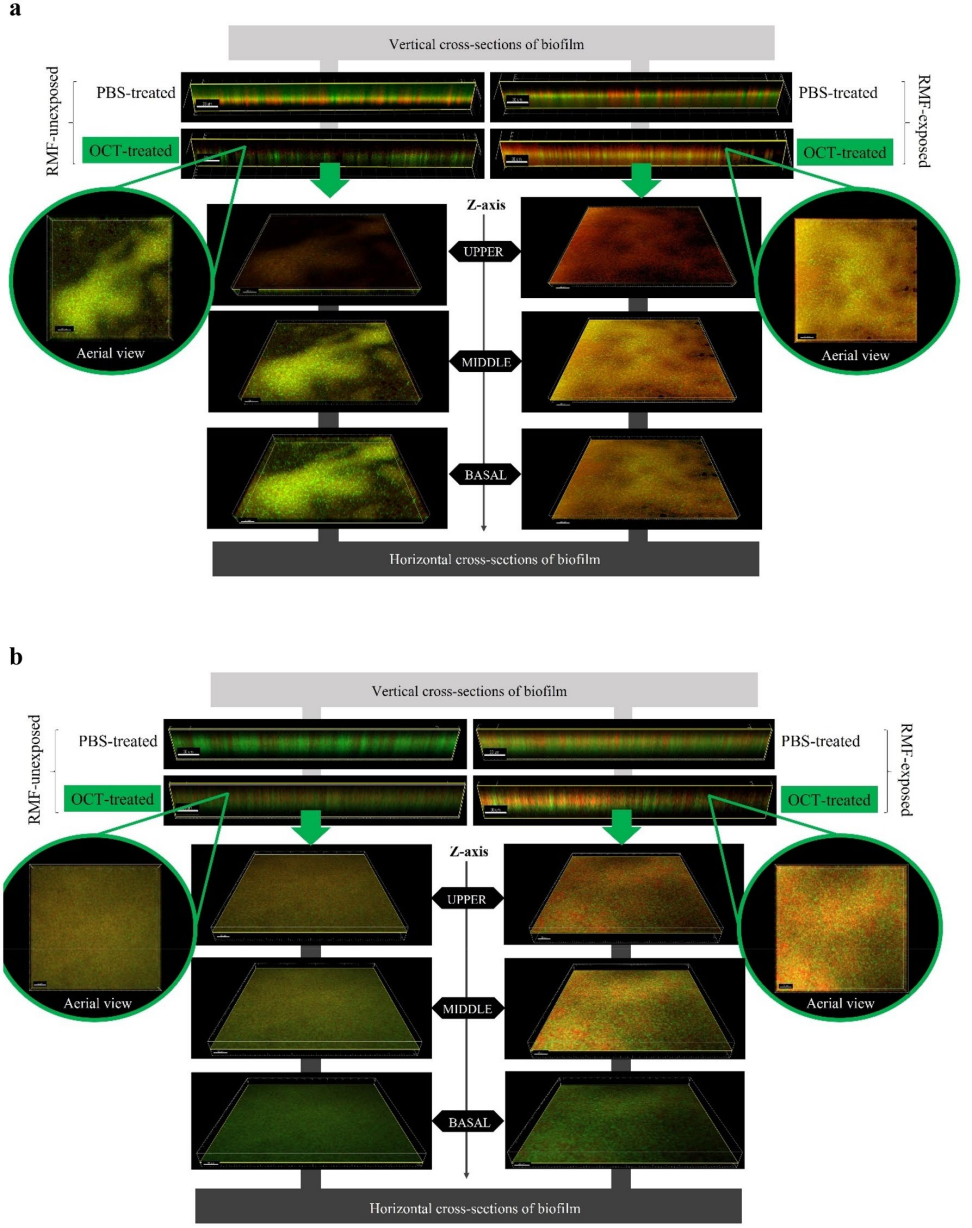
The spatial location of (**a**) staphylococcal and (**b**) pseudomonal cells within biofilm treated with OCT-saturated carriers and/or exposed to RMF (5 Hz). The cells with non-altered cell walls dye green (as a result of SYTO-9), while the cells with compromised cell walls dye red/orange (as a result of propidium-iodide incorporation).

The application of the RMF regardless of its frequencies increased the number of *S. aureus* and *P. aeruginosa* cells with altered (compromised) cell walls (Fig. 7; since no substantial differences were observed between the applied RMF frequencies, the results obtained for 50 Hz are presented in Supplementary Information, Supplementary Fig. S7). Comparable changes were observed across the whole structure of biofilm (from the upper, through the middle to the basal layers). Likewise, the incubation of biofilms with OCT-saturated carriers together with the exposure to RMF resulted in a significantly higher level of destructed cells (also in the whole cross-section of biofilm) compared to biofilms incubated with OCT-saturated carriers but unexposed to the RMF.

**Figure 8:**
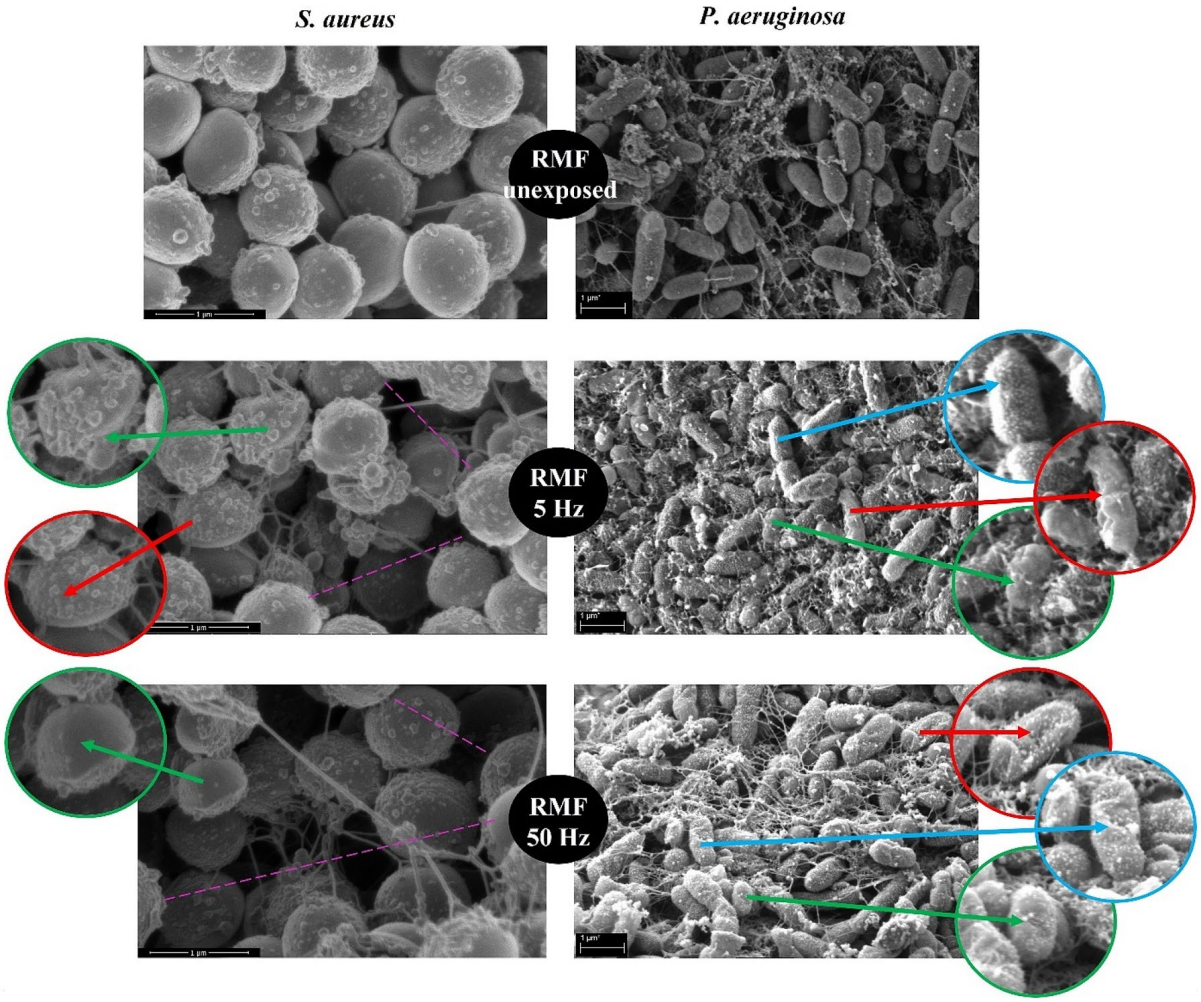
Morphology of (**a**) staphylococcal and (**b**) pseudomonal cells within biofilm structure exposed and unexposed to RMF. Green arrows indicate changes in cellular morphology; the red arrows indicate loss of turgor; blue arrows indicate granularities on the cell surface. The purple lines point out the increased distance between cells in biofilms exposed to the RMF. The magnification of staphylococcal biofilm equals 50,000× and pseudomonal biofilm 30,000×.

**Figure 9:**
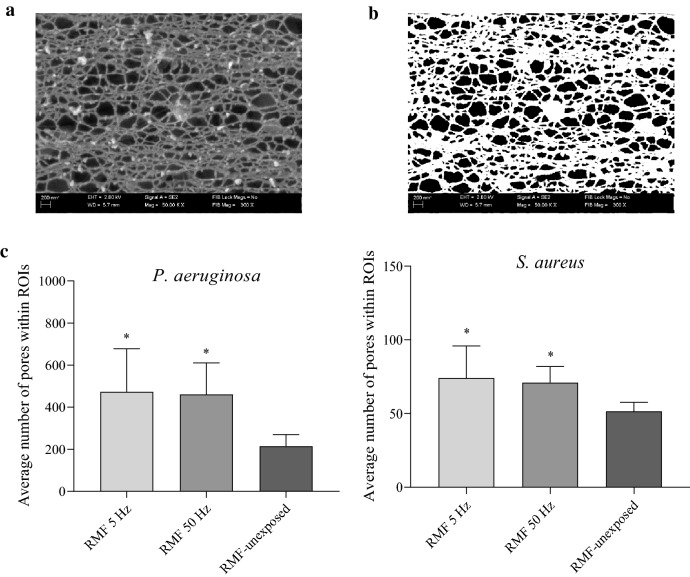
The porosity of biofilm matrix exposed and unexposed to RMF. (**a**) image of representative *P. aeruginosa* biofilm matrix, (**b**) image processing of matrix leading to differentiation of fibrils (white shapes) from matrix pores (black shapes), (**c**) average number of pores in *S. aureus* and *P. aeruginosa* biofilm matrix exposed and unexposed to RMF. The results are presented as mean ± SEM. The asterisks in graphs indicate statistical significance (*p* < 0.05) between respective average values.

Because the results of the confocal microscopy indicated that the RMF can alter cell walls of biofilm-forming cells, in the next step of investigation we analyzed the character of these changes using the SEM. As can be seen in Fig. 8, the application of the RMF (of both 5 Hz and 50 Hz frequencies) correlated with a spectrum of changes in biofilm-forming cells of *P. aeruginosa* and *S. aureus*. These alterations included loss of turgor, a change of shape (particularly well-seen in *P. aeruginosa* cells); in the case of *S. aureus*, the increase in distance between cells was also observed. Following this last observation, we performed a parametric analysis of the RMF-induced changes in the pseudomonal and staphylococcal biofilm matrix. The results presented in Fig. 9 indicated that the number of pores in *S. aureus* and *P. aeruginosa* biofilm matrices were statistically higher (*p* < 0.05) compared to the RMF-unexposed biofilms. In turn, the differences between the number of pores in biofilms exposed to the RMF of 5 or 50 Hz, were statistically insignificant. The average pore number in *S. aureus* biofilm matrix, exposed to the RMF of 5 or 50 Hz, was 43% and 37% higher, respectively, compared to the RMF-unexposed biofilms. In the case of *P. aeruginosa* these values were 220% and 214%, for 5 and 50 Hz exposure respectively.

### Influence of RMF on biofilm matrix composition

In subsequent analyses, we assessed the impact of the RMF on the biofilm matrix with regard to its molecular composition. Before the analysis, the cells and their leftovers were chemically removed from the biofilm; the effectiveness of this procedure was confirmed using the cryo-SEM technique and then, the cell-free matrix of biofilms exposed or unexposed to the RMF was subjected to GC–MS/MS analyses (Supplementary Fig. S8).

The GC–MS/MS analysis was targeted to sugars, considered one of the major components (in a form of polysaccharides) of both pseudomonal and staphylococcal biofilm matrices. It was found that the application of the RMF (5 and 50 Hz) altered the saccharide composition of both analyzed biofilms (Table 1). In the case of *P. aeruginosa*, a distinct trend was observed, i.e. the content of 11 (out of 13 detected) saccharides (and their isomers) was lower in the RMF-exposed biofilm compared to the RMF-unexposed setting; moreover, the drop of content in these 11 saccharides was stronger for the RMF of 5 Hz comparing to the RMF of 50 Hz. The saccharides which did not fit the above pattern were b-fucose and a-galacturonic acid. In the case of fucose, exposure to the RMF increased its content in comparison to the RMF-unexposed setting (to a higher extent at 50 Hz than at 5 Hz), while in the case of a-galacturonic acid, its content was basically the same in the matrix obtained from biofilm unexposed vs. exposed to the RMF 50 Hz (0.15 vs. 0.14 ng/mg, respectively) and higher (0.15 vs. 0.23 ng/mg) due to the exposure to the RMF of 5 Hz. In the case of the staphylococcal matrix, the drop of saccharide content was observed for 4 (out of 15 detected) saccharides: *a*-arabinose, *b*-rhamnose, *a*-fucose, and *b*-fucose. The content of the remaining 11 saccharides was higher in the matrix obtained from biofilm exposed to the RMF as compared to the RMF-unexposed setting; in the case of 7 (out of 11 saccharides matching the above pattern), the increase was higher in biofilm exposed to the RMF of 5 Hz comparing to the RMF of 50 Hz.

**Table 1:** The average content of specific sugars [ng/mg of sample] in cell-free *S. aureus* and *P. aeruginosa* matrix obtained from biofilms exposed and unexposed to RMF.

Sugar [ng/mg]	*S. aureus*			*P. aeruginosa*		
	RMF-unexposed	RMF 5 Hz	RMF 50 Hz	RMF-unexposed	RMF 5 Hz	RMF 50 Hz
*a*-Arabinose	2.55 ± 0.24	1.68 ± 0.07	1.20 ± 0.12	0.32 ± 0.08	0.13 ± 0.04	0.27 ± 0.04
*b*-Arabinose	6.41 ± 0.32	8.22 ± 0.14	7.66 ± 0.72	0.76 ± 0.12	0.48 ± 0.16	0.60 ± 0.13
*a*-Galactose	5.56 ± 0.43	6.78 ± 0.31	7.65 ± 1.53	0.92 ± 0.2	0.25 ± 0.05	0.41 ± 0.06
*b*-Galactose	15.43 ± 0.56	17.40 ± 2.26	17.59 ± 3.62	0.00	0.00	0.00
*a*-Glucose	64.03 ± 3.56	79.24 ± 7.32	71.27 ± 7.07	1.88 ± 0.2	0.92 ± 0.21	1.57 ± 0.25
*b*-Glucose	33.89 ± 3.10	41.31 ± 4.34	40.23 ± 8.09	3.01 ± 0.14	0.91 ± 0.31	2.02 ± 0.31
*a*-Mannose	8.31 ± 0.14	12.33 ± 1.78	10.01 ± 0.61	2.95 ± 0.06	0.71 ± 0.17	1.78 ± 0.09
*b*-Mannose	15.44 ± 0.67	17.45 ± 1.67	17.63 ± 2.68	2.58 ± 0.2	1.21 ± 0.32	2.20 ± 0.27
*a*-Rhamnose	1.31 ± 0.21	3.10 ± 0.23	2.30 ± 0.34	3.92 ± 0.14	1.13 ± 0.07	2.82 ± 0.15
*b*-Rhamnose	3.67 ± 0.45	2.55 ± 0.67	1.31 ± 0.44	3.35 ± 0.8	2.19 ± 0.11	3.42 ± 0.03
*a*-Galacturonic acid	2.31 ± 0.34	2.42 ± 0.30	2.70 ± 0.61	0.15 ± 0.04	0.23 ± 0.02	0.14 ± 0.07
*b*-Galacturonic acid	9.89 ± 0.34	12.78 ± 0.31	11.87 ± 1.22	0.00	0.00	0.00
*N*-Acetyl-glucosamine	897.03 ± 33.29	1034.11 ± 145.45	952.12 ± 44.8	0.21 ± 0.04	0.34 ± 0.11	0.21 ± 0.10
*a*-Fucose	2.20 ± 0.42	1.01 ± 0.01	1.05 ± 0.30	0.00	0.00	0.00
*b*-Fucose	80.45 ± 3.02	75.56 ± 4.31	71.91 ± 12.83	0.12 ± 0.01	0.16 ± 0.03	0.33 ± 0.07
*a*-Xylose	0.00	0.00	0.00	1.62 ± 0.37	0.78 ± 0.14	0.98 ± 0.22
*b*-Xylose	0.00	0.00	0.00	1.12 ± 0.33	0.46 ± 0.01	0.90 ± 0.27

## Discussion

The current study aimed to determine the factors standing behind the observed increased antibiofilm efficacy of octenidine dihydrochloride-based antiseptic in the presence of the RMF. We assumed, that the increased effectiveness of the antiseptic in the presence of RMF should be interpreted as the appearance of the antibiofilm effect observed in a shorter contact time and/or with the concentration of the active substance, which did not cause any bactericidal effect if the RMF was not applied. For this reason, we used the A.D.A.M. test, previously developed by our team^22^, dedicated to the determination of the antibiofilm effect exerted by the active substance, penetrating the three biofilm structures separated by three porous obstacles (represented by 3 agar discs in test). Moreover, for the purposes of this study, it was necessary to apply such antiseptic dilution that displayed any or scanty antibiofilm effect during the particular (experimental) contact time but caused an antibiofilm effect if the contact time was extended. Such assumption was taken, based on the mechanism of action of octenidine dihydrochloride-based antiseptics, which (even when diluted), can cause a bactericidal effect if a sufficiently long contact time is provided^24^. Following the same thinking pattern, also the selected exposure time to the RMF (3 h), was too short to change significantly the viability of the biofilm-forming cells. Thus, if the antibiofilm effect occurred, it would be a result of the simultaneous effects displayed by the antiseptic and the RMF. For this reason, in the first stage of the study, it was confirmed that the application of antiseptic-saturated carrier did not translate into a drop of viable biofilm-forming cells of both *S. aureus* and *P. aeruginosa* bacterial species, on any of the agar discs (C, M, B) during the particular 3 h contact time. Simultaneously, the application of the same (with regard to antiseptic concentration) carriers reduced the percent of viable biofilm-forming cells when the contact time was exceeded over a 3 h period. The reduction in the number of live bacterial cells was particularly visible only in the case of biofilms incubated with antiseptic solutions for 24 h, which was most likely related to the relatively low concentration of the active substance applied solution and the acknowledged prolonged mechanism of the antiseptic action^24,25^. In addition, lower values of cell viability were found for *S. aureus* as compared to *P. aeruginosa*, which in turn could be related to the differences in the structure and composition of the biofilm matrix produced by these bacterial species. As shown in the SEM analysis, the *P. aeruginosa* biofilm contained significantly greater amounts of the protective matrix than *S. aureus* biofilm. Moreover, the biofilm matrix of *P. aeruginosa* contains not only exopolysaccharides^26,27^ but also phospholipids, which are one of the main target sides of octenidine hydrochloride molecules^25^, which may explain the observed differences in this antiseptic activity against biofilms formed by two different bacterial species.

The literature data indicated that various types of magnetic fields may influence the number of live bacterial cells, causing the reduction^28–31^ or increase^32–34^ in their number. There are also numerous studies where the application of magnetic fields did not alter the number of exposed bacteria^35–38^. It is widely recognized that the effect of the magnetic field is to a major extent related to its characteristics (e.g. frequency, intensity, and distribution of magnetic field lines) and time of exposure. Therefore, in our research, we analyzed the effect of exposure to the RMF of different frequencies (5 and 50 Hz) for 1, 2, and 3 h on changes in the viability of bacteria present on each of the agar discs in applied biofilm model. However, it was found, that the exposure of biofilm samples to the RMF (without the antiseptic) did not significantly affect the viability (measured by standard metabolic tests) of *S. aureus* and *P. aeruginosa* cells regardless of the exposure duration and RMF frequencies. There were also no statistically significant differences in the viability of RMF-exposed cells forming a biofilm on the specific (C, M, B) agar discs. In our previous studies, we showed that the RMF affected the cell viability of *S. aureus* and *P. aeruginosa*, but it has to be underlined that these studies were conducted for bacteria cultured in liquid cultures, during their phase of logarithmic growth^18^. In the present study, the exposed bacteria were immobilized on the surface of agar discs and anchored within the biofilm matrix. As it is commonly known, bacterial biofilm communities are characterized by a much greater resistance to all stressors (including antimicrobials) as compared to the same bacteria in liquid suspension (referred to as the planktonic phenotype)^39–41^. The access of biofilm-forming bacteria to nutrients and oxygen (in the case of aerobic species) is limited and thus regulated by the biofilm community itself by complex pathways of information exchange^42^. In this context, it can be said, that one of the factors limiting the action of antimicrobial substances is the decreased metabolic activity of cells in the biofilm^39^. Thus, it can be assumed that bacteria with lower activity are also less sensitive not only to chemical agents (e.g. antibiotics and antiseptics) but also to physical factors (e.g. magnetic fields). It is also worth emphasizing that it is for this reason that in the conducted analyzes we chose the MTT test, which in addition to its standard purpose (analysis of cell viability) provided us also data on changes in cellular metabolic activity^43^. On the other hand, it should also be noted that in the current study, due to the relatively short experimental time selected for the analysis of the antimicrobial effect induced by antiseptics, exposure to the RMF was also relatively short as compared to our earlier research^19^, as well as compared to the research by other authors^10,12^.

In the further stages of the analysis, it was also shown, that the exposure of biofilm samples to the RMF (regardless of its frequency) when combined with the solution of the antimicrobial, significantly increased the antibacterial effect against *S. aureus* as well as *P. aeruginosa,* although in the case when both factors acted separately, no effect (measured by the MTT test) was observed. The effect was mainly dependent on the time of exposure to the RMF and to a lesser extent on the biofilm-forming bacterial species and the RMF frequency. Such observation stays in line with the previous reports of our research group^17,44^ as well as reports of other authors^13,45^ indicating that the time of magnetic exposure is also of key importance concerning the effect exerted on biological systems. Depending on the exposure time, magnetic fields may have a different effect on bacterial viability, i.e. it may increase it^32–34^ or reduce it^28–31^.

It was also found that the RMF influenced the degree of octenidine dihydrochloride penetration into specific agar discs. The greatest effect was observed during continuous, 3 h exposure to the RMF and stronger for frequency of 50 Hz than of 5 Hz (the difference exceeded 20%). When the experimental setting was exposed for a time shorter than 3 h, the concentration of octenidine dihydrochloride was also higher, compared to the RMF unexposed setting, however this time, the differences did not exceed 3%. As already mentioned, the strength of magnetic field impact (regardless of its type or the phenomenon analyzed in its presence), except for the exposure duration, depends on its intensity and/or frequency^17,28,44,46^, because these two factors determine the physical characteristics of the magnetic signal^47,48^. In the case of the RMF setup used in the present study, the magnetic field is axially symmetric and magnetic field lines rotate in a horizontal direction, with the rotation frequency equal to the frequency of the electric current^49^. Therefore, depending on the applied electric current frequencies, also the synchronous speed of the magnetic flux rotation around the stator is different, as can be seen in the simulations: 5 Hz—https://www.youtube.com/watch?v=EwojY3mR11A; 50 Hz—https://www.youtube.com/watch?v=swCPxvRdZoc.

**Figure 10:**
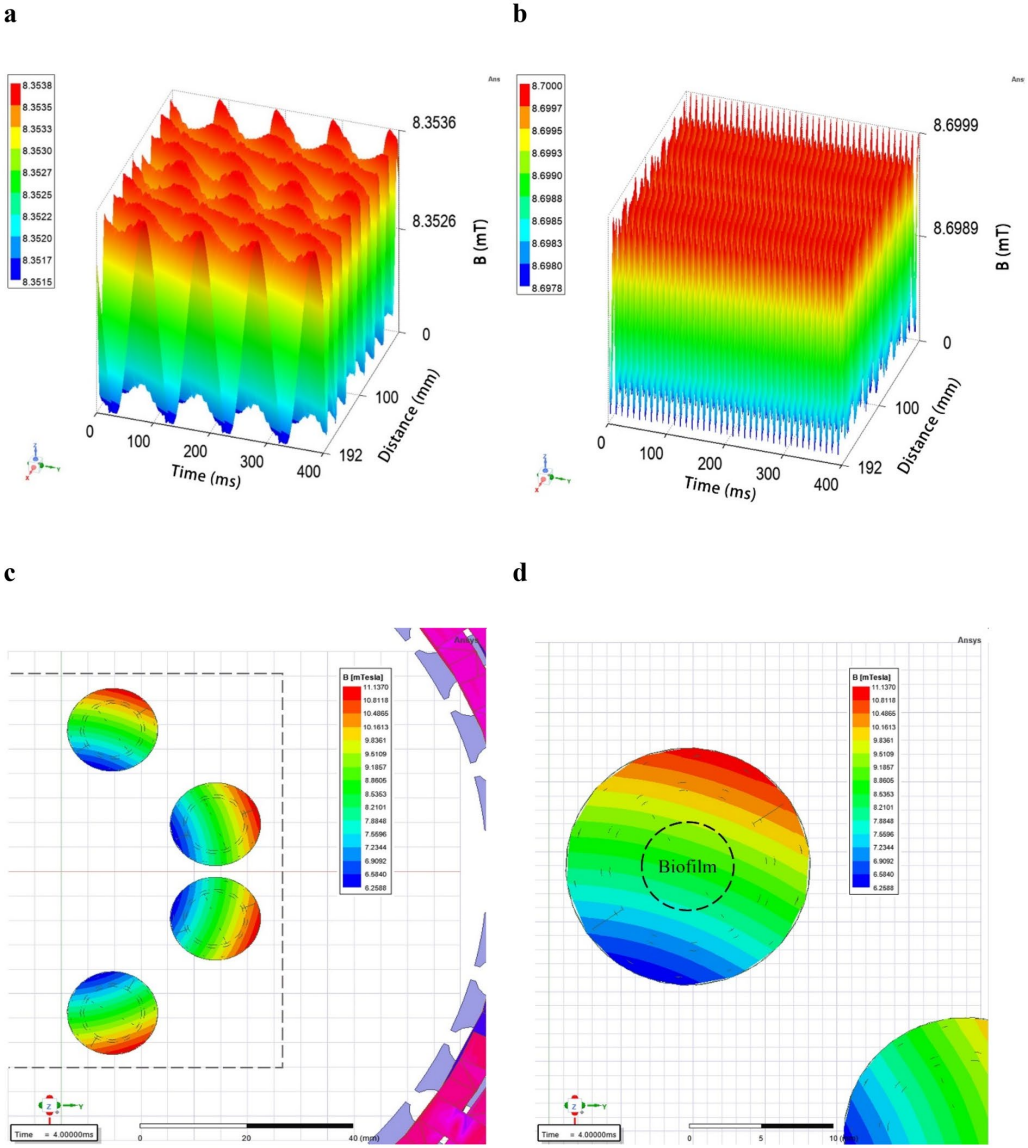
Periodical changes in magnetic induction in the center of the well of a test plate depending on the applied current frequency: (**a**) 5 Hz; (**b**) 50 Hz and distribution of the magnetic induction: (**c**) in the wells of a test plate; (**d**) at the location of the agar disc covered with biofilm.

Moreover, the electric current frequency determines the magnetic field intensity and it is responsible for the magnetic wave's physical characteristics. At 5 Hz (the lowest current frequency that can be used in the setup), the amplitude of the RMF was characterized by a longer period of maximal/minimal magnetic induction (*B*) state with *B*_max_/*B*_min_ 8.354/8.352 mT. In contrast, the RMF generated at 50 Hz (the highest current frequency that can be used in the setup) was characterized by a shorter period, with *B*_max_/*B*_min_ 8.700/8.698 mT (Fig. 10, Supplementary Table S2). It was also estimated, that the observed effects resulted mainly from the influence of the magnetic field, whereas the energy flux density affecting the exposed samples caused by the generated electric field was negligible. The estimation of the energy flux density of the electric field is presented in Supplementary Information.

Nevertheless, although our studies showed that the RMF influence the rate of octenidine dihydrochloride penetration, it should be noted that these differences should not be treated as the fundamental reason for the changes observed in biological analyzes using bacterial biofilms (A.D.A.M. test). In these analyzes, as already mentioned, the applied antiseptic concentration, (6.25% for *S. aureus* and 25% for *P. aeruginosa*) did not reduce the viability of the bacterial cells (in case of the contact time of 3 h). Therefore, taking into account approx. 25% increase in the concentration of the octenidine dihydrochloride as a result of exposure to the RMF for 3 h, it could be also assumed that the results obtained in the A.D.A.M. could be bound to the higher concentration of the active substance in the agar discs. However, the analyzes carried out using the LC–MS/MS technique did not show any substantial differences in the octenidine dihydrochloride concentrations as a result of exposure to the RMF for 2 h as compared to the exposure lasting for1 h. In turn, the results obtained from the measurement of the antibiofilm activity using the A.D.A.M. test did not differ significantly between 3 and 2 h exposure variants. Thus, although the 2 h RMF-exposed agar discs contained approximately the same concentration of octenidine dihydrochloride as the discs exposed for 1 h, the antimicrobial effect recorded after the exposure lasting for 2 h was comparable to the one which was observed after 3 h. For this reason, it can be assumed that although the increased concentration of octenidine dihydrochloride may have a positive impact on the outcome observed, the interpretation of the results should explicitly take into account also the influence of the RMF on bacterial cells and/or the biofilm matrix. With regard to that, the previous studies have indicated the ability of different types of magnetic fields to disturb microbial structures, i.e. to permanently damage the bacterial cell walls, presumably by their irreversible electroporation^50,51^. Fojt et al. (2004) explained that a drop in bacterial viability after exposure to the magnetic field was caused by an increase in the permeability of ion channels in the cytoplasmic membranes or by the formation of free radicals in bacterial cells^28^. Similarly, also in our previous studies concerning the impact of combined use of the RMF with various classes of antibiotics against *S. aureus*, the most promising results involved two groups of antibiotics, β-lactams, and glycopeptides^20^, the common feature of which is the site of their antimicrobial activity, i.e., the bacterial cell wall.

The in vitro biofilm, applied in our research consists of three components: biofilm-forming cells, biofilm matrix, and biofilm milieu understood here as the medium immersing biofilm and filling the empty spaces (the light of matrix pores) within biofilm structure. The analyses of the distribution of biofilm-forming cells and the integrity of their cell walls, performed using confocal microscopy indicated at least two noticeable phenomena. The first of them was that number of cells with altered/compromised cell walls in biofilms exposed to the RMF only (without the antiseptic) was higher compared to the unexposed controls; while the second of them was that these cell-wall altered cells were found in every analyzed (upper, middle and basal) biofilm cross-section. Bearing in mind the fact that metabolic analyses indicated no loss of cell viability, it has to be assumed that alterations of cell walls being the result of the RMF activity were of minor functional character (they did not lead to cell death, but at the same time they were strong enough to be detected by applied visualization technique). In turn, the occurrence of altered cells across the Z-axis of the whole biofilm structure suggests that this effect should be attributed to the RMF, and its wave-like properties, not compromised by such physical factors as biofilm thickness or matrix density. Noteworthy, the data obtained from confocal microscopy showed high compliance with the results of metabolic tests with this regard that combined application of the RMF and antiseptic led to the strong eradication of biofilm in all its parts, while the application of the antiseptic only (in a given 3 h contact time) did not correlate with a significant drop of viable cells and with the increase of dead cells.

The subsequent analysis performed using SEM revealed that the RMF-exposed cells forming pseudomonal or staphylococcal biofilm exhibit the whole spectrum of alterations, recognized as typical manifestations of disturbances in cell walls/membranes^52^. Even weak magnetic fields, such as the RMF applied in the current work, are considered to be able to cause not only the already-mentioned mixing effect of electrically charged particles in liquids or colloids (such as bacterial cytoplasm containing large quantities of mobile ions) but also to excite the electrons in cytoplasmatic molecules. As shown by Hong et al. (1995), the effects of such magnetic induction concentrate on the cellular walls/membranes, due to the shielding effect mostly^53^. Such a statement is coherent not only with the data presented in the current work (showing disturbances in the bacterial cell walls as a result of the RMF exposure) but also with the results shown recently by Sharpe et al. (2021), who reported the damage of mitochondrial membranes in cancer cells exposed to the RMF^54^. It should be underlined that process of biofilm matrix formation is facilitated to a major extent by specialized protein systems localized throughout the bacterial cell walls and membranes^55^. Because the impact of the RMF on these particular cellular compartments was explicitly shown (and partially elucidated) in this work, it would be rational to assume that their alteration may have also an impact on the secreted biofilm matrix. Indeed, the average number of pores in the RMF-exposed biofilm matrices was significantly higher as compared to the RMF-unexposed controls (regardless of the species). We are aware of fact that such parametric analysis of SEM images, as performed, might be not completely representative, taking into consideration the fact that only a relatively small area of the whole biofilm structure could be analyzed by its means. Therefore, this part of the experiment should be treated as the pilotage and it requires the development with the use of more suitable, parametric analyses. To overcome the above-mentioned disadvantage, we performed GC–MS/MS analyses of the whole, cell-free biofilm matrices to investigate whether the application of the RMF translates into a change of one of their main components, i.e. saccharides. The results showed that the concentration of all detected saccharides changed in the RMF-exposed biofilms (regardless of the applied RMF frequency and biofilm-forming bacteria). In the case of the Gram-negative *P. aeruginosa*, the significant drop of saccharides’ concentration (in the setting applying the RMF of 5 Hz, mostly) was visible, while such an effect was not detected so explicitly in the case of the Gram-positive *S. aureus* biofilm. Taking into consideration the data presented in the already mentioned work of Hong et al. (1995) concerning the shielding effect manifesting primarily in cellular boundaries, it may be assumed that the rigid, multi-layer cell wall of *S. aureus* was more resistant (than relatively thin cell wall of *P. aeruginosa*) to the RMF-induced electron excitation and resulting disturbances in the protein-mediated secretion of matrix components^53^. Such observation requires, undoubtedly, further investigation of the biofilm proteome (with special stress put on the membrane/cell walls proteins) to interconnect the already demonstrated changes in the biofilm matrix with possible alteration in these proteins’ expression and concentration. Moreover, in our analyses, we focused on the saccharides only, while recent data show that also other components, such as extracellular DNA may constitute the majority of the biofilm matrix^56^. Nevertheless, even at the present stage, the presented data is another example of the complex nature of the interaction of RMF on biofilm structure.

When octenidine dihydrochloride-based antiseptic is applied against pathogenic biofilm, it needs to, in the first place, penetrate the biofilm milieu. In the case of the experimental model applied in this research, the milieu is microbiological medium and agar discs. As we already indicated in our earlier work^2^, the plethora of organic compounds in applied TSB medium, derived from hydrolyzate of casein and soybean, may have an impact on cationic octenidine dihydrochloride. In fact, the normative methods of assessment of antiseptic activity imply the use of an “organic burden” to analyze the impact of this load on antiseptic molecules' activity, which is mostly negative (the effectiveness drops)^57^. The agar discs are another component of the biofilm milieu. The parametric processing of SEM images of agar surfaces revealed that the average Ferret’s diameter of agar pores, through which octenidine dihydrochloride molecules had to penetrate was 70 nm (Supplementary Fig. S9), which is coherent with the data presented by Crowle et al. (1973)^58^. Such pore size, at the first glance, should not interfere with the penetration of a relatively small molecule (106 atoms) of octenidine dihydrochloride. Nevertheless, one-third of agar consists of agaropectin, which is negatively charged due to the presence of pyruvate and sulfate groups^59^. Therefore, one may assume that such negatively charged, medium-filled nano-pores of agar may interact with the cationic (i.e. net positive) charge of octenidine dihydrochloride and decrease its penetrability. The antiseptic needs also to penetrate through the biofilm matrix, which may consist of molecules trapping its molecules and decreasing its concentration. Next, the octenidine dihydrochloride binds to the cell walls and membranes and disrupts them, leading to the cells’ death followed by leakage of cytoplasm to the external environment.

In turn, when RMF is applied as a self-reliant factor, it interacts with virtually all components (milieu, biofilm matrix, and the biofilm-forming cells) of the applied experimental model. The RMF increases also the penetration of cationic octenidine dihydrochloride molecule through the agar pores-filling medium, by increasing its mixing efficacy. The RMF-induced mixing effect occurs also in intracellular, colloidal cytoplasm which exerts elevated pressure on bacterial cell walls^15,60^. Another RMF-induced phenomenon is related to supra-molecular excitation of electrons^55^, manifesting mostly in, indicated in this work, disturbances of cell walls/membranes. These alterations are also most likely the reason standing behind observed changes in the biofilm matrix porosity and composition because they affect the cell wall/membrane-localized protein systems facilitating synthesis and transportation of matrix components from the cell to the external environment. Although all these above-mentioned RMF-induced changes, seem to not disturb significantly the general viability of the staphylococcal and pseudomonal biofilms (as indicated in metabolic tests), they significantly facilitate the activity of antiseptic by weakening the cell wall/membrane structures (main target site of the octenidine dihydrochloride). From the clinical perspective and future potential application of the RMF as a therapeutic agent, it can be assumed that thanks to the RMF it is possible to destroy the biofilm, even in its basal layers, with the use of relatively low concentrations of antiseptic, in a much shorter contact time, comparing to the setting in which the same therapeutical effect would be achieved, but without the use of the magnetic field.

In conclusion, the current study showed, that 3 h exposure to the RMF without antiseptic did not destroy biofilm as compared to the unexposed controls. Similarly, the application of antiseptic without the RMF did not influence biofilm viability during 3 h contact time. However, in the simultaneous presence of the antiseptic and RMF, the significant antibiofilm effect was revealed after already 1 h of the exposure, and it increased along with the exposure time. It was also demonstrated, that the RMF weakened the cell wall/membranes of biofilm-forming, increased the porosity of the biofilm matrix, and altered its chemical composition. Therefore, in the future perspective, the RMF may find an application as a therapeutic agent increasing antiseptics’ effectiveness, especially in an environment where physical obstacles may hinder their activity.

## Supplementary Information

Supplementary Information.

## Data Availability

The original datasets generated during the current study have been deposited with link to Figshare database (https://figshare.com/articles/dataset/The_Multi-Directional_Effect_of_Rotating_Magnetic_Field_and_Antiseptic_on_In_Vitro_Pathogenic_Biofilm_and_Its_Milieu/19494143).
