# Supplementary material for: The effects of rotating magnetic field and antiseptic on in vitro pathogenic biofilm and its milieu
Source: Sci Rep. 2022 May 25;12:8836. doi: 10.1038/s41598-022-12840-y (PMC9132948; doi:10.1038/s41598-022-12840-y)
Supplement: Supplementary file 1 — Supplementary Information. [file 41598_2022_12840_MOESM1_ESM.docx]

**SUPPLEMENTARY INFORMATION**

**The Effects of Rotating Magnetic Field and Antiseptic on In Vitro Pathogenic Biofilm and Its Milieu**

**Daria Ciecholewska-Juśko^1^, Anna Żywicka^1^, Adam Junka^2,*^, Marta Woroszyło^1^, Marcin Wardach^3^, Grzegorz Chodaczek^4^, Patrycja Szymczyk-Ziółkowska^5^, Paweł Migdał^6^, Karol Fijałkowski^1,*^**

^1^ Department of Microbiology and Biotechnology, Faculty of Biotechnology and Animal Husbandry, West Pomeranian University of Technology, Szczecin, Piastów 45, 70-311 Szczecin, Poland

^2^ Department of Pharmaceutical Microbiology and Parasitology, Faculty of Pharmacy, Medical University of Wroclaw, Borowska 211a, 50-534 Wrocław, Poland

^3^ Faculty of Electrical Engineering, West Pomeranian University of Technology, Szczecin, Sikorskiego 37, 70-313 Szczecin, Poland

^4^ Laboratory of Confocal Microscopy, Łukasiewicz Research Network-PORT Polish Center for Technology Development, Stabłowicka 147, 54-066 Wrocław, Poland

^5^ Centre for Advanced Manufacturing Technologies (CAMT/FPC), Faculty of Mechanical Engineering, Wroclaw University of Science and Technology, Łukasiewicza 5, 50-371 Wrocław, Poland

^6^ Department of Environment, Hygiene and Animal Welfare, Faculty of Biology and Animal Science, Wroclaw University of Environmental and Life Sciences, Chełmońskiego 38C, 51-630 Wrocław, Poland

* [karol.fijalkowski@zut.edu.pl](mailto:karol.fijalkowski@zut.edu.pl); [adam.junka@umed.wroc.pl](mailto:adam.junka@umed.wroc.pl)

**Table S1.** The main data of the RMF generator.

| **No.** | **Parameter** | **Description** | **Value** |
| --- | --- | --- | --- |
|  | *R_so_* | Stator’s outer radius | 110.0 mm |
|  | *R_si_* | Stator’s inner radius | 80.0 mm |
|  | *l* | RMF generator’s length | 199.0 mm |
|  | *p* | Number of pole pairs | 2 |
|  | *k_s_* | Number of stator windings in slot | 50 |
|  | *s* | Number of slots | 36 |
|  | *U_n_* | Nominal phase voltage | 230 V |
|  | *I_n_* | Nominal phase current | 17.1 A |
|  | *p_b_* | Number of winding parallel branches | 2 |
|  | *R_ph_* | Phase resistance | 1.94 Ω |
|  | *L_ph_* | Phase inductance | 8.04 mH |

RMF generator is powered by an inverter with the use of U/f = const control. The stator used was from a mass-produced 15 kW four-pole induction motor (INDUKTA S.A., 2SIE160L4, Poland). Since the stator worked without a rotor (there were test samples in place of the rotor), the nature of its operation, from the point of view of power supply, was similar to that of a three-phase choke. As a result, the induced voltage (back-EMF) reached a relatively small value, therefore it was necessary to limit the supply voltage to a value of 100 V. The stator used is a three-phase wound and star-connected. After applying power to the stator phase bands, magnetic poles were formed inside the stator, which shifted in time according to the frequency of the supply current. Thus, a system configured in this way generated a rotating magnetic field (RMF) inside the stator.

| **a** | **b** |
| --- | --- |
|  |  |

**Figure S1.** The antibiofilm effect of OCT solution after 3 h contact time, depending on the dilution, (**a**) *S. aureus* and (**b**) *P. aeruginosa.*

| **a** |
| --- |
| **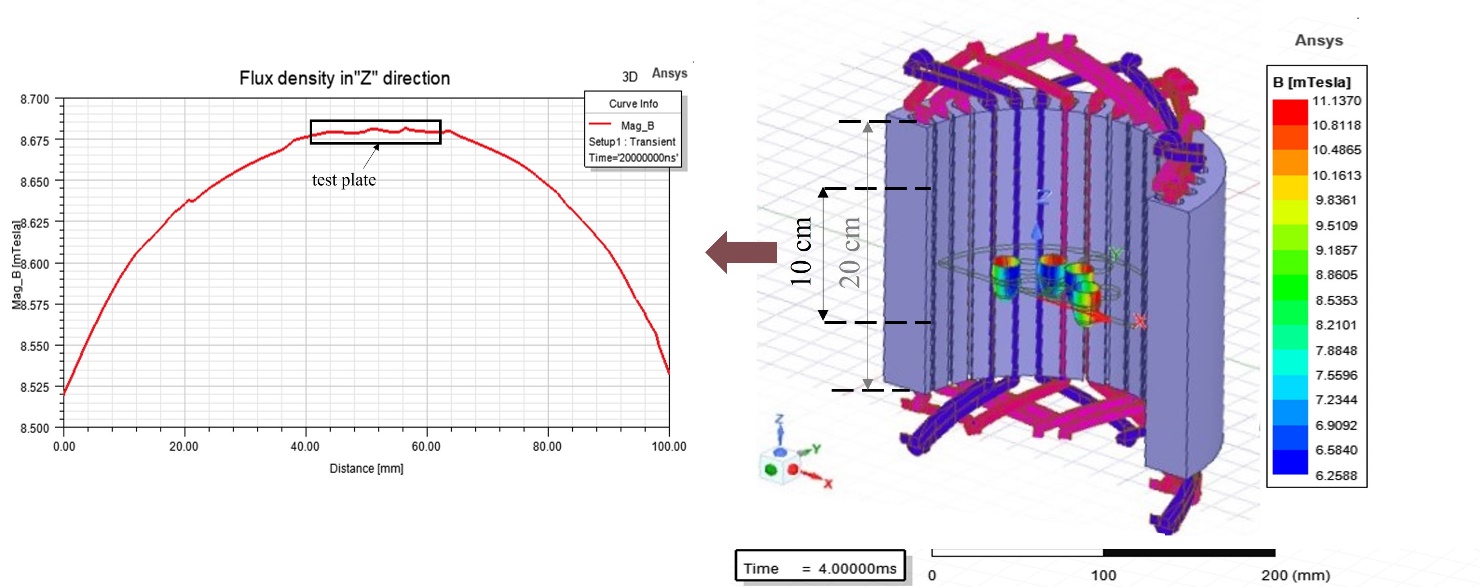** |
| **b** |
| 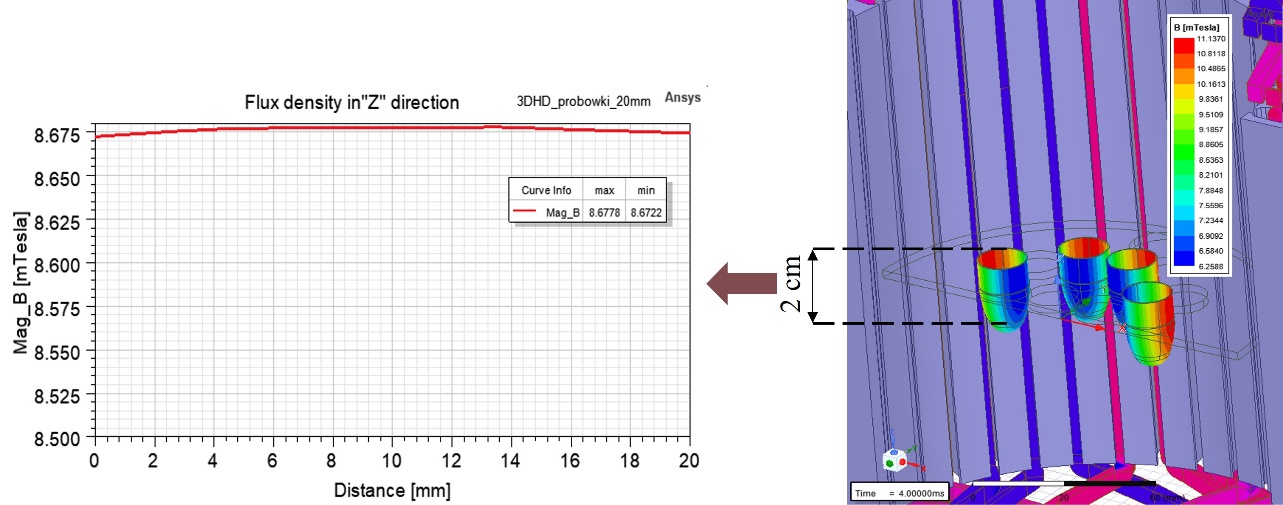 |

**Figure S2.** Spatial distribution of magnetic field a) in cross-section (h = 10 cm) of RMF-bioreactor in center of representative well of a test plate, b) in cross-section of center of representative well of a test plate (h = 2 cm) placed inside RMF-bioreactor.

**
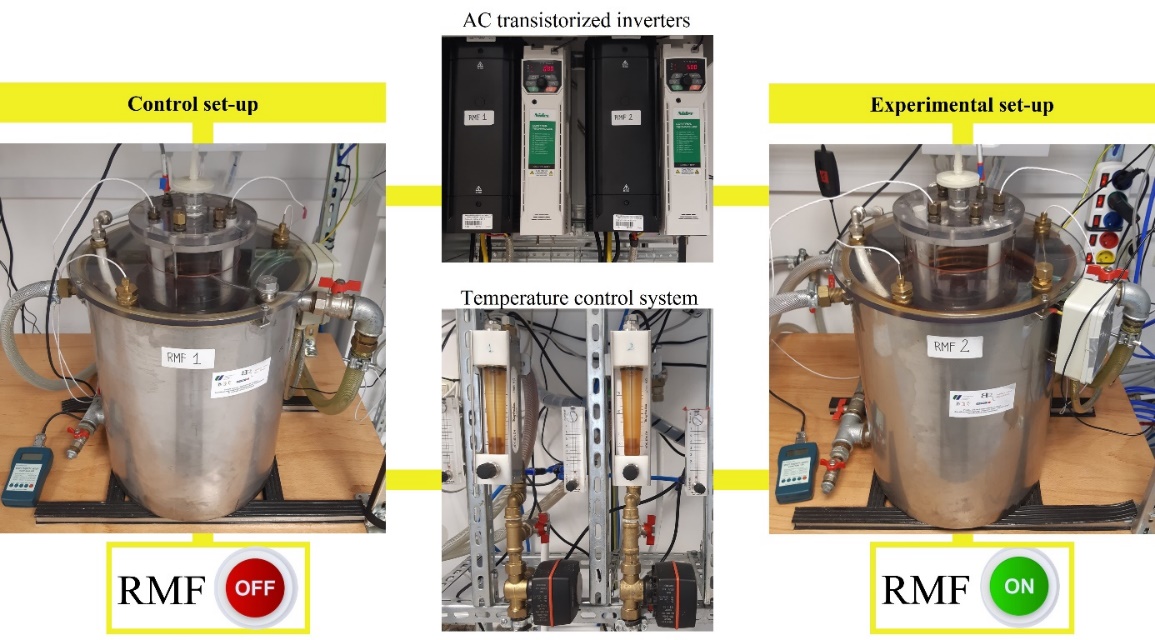
**

**Figure S3.** RMF generator and control settings with monitoring and control equipment.

| **a** | **b** |
| --- | --- |
|  |  |
| **c** | **d** |
|  |  |

**Figure S4.** Percent of living biofilm-forming cells on successive agar discs exposed to RMF of (**a**) *S. aureus* RMF of 5 Hz, (**b**) *S. aureus* RMF of 50 Hz, (**c**) *P. aeruginosa* RMF of 5 Hz and (**d**) *P. aeruginosa* RMF of 50 Hz in comparison to RMF-unexposed settings.

The results are presented as mean ± SEM. There were no statistically significant differences in cells viability between individual discs (C, M, B) and in comparison to RMF-unexposed control (p < 0.05, Tukey’s HSD test).

| **a** | **b** |
| --- | --- |
|  |  |

**Figure S5.** Percent of living (**a**) *S. aureus* and (**b**) *P. aeruginosa* biofilm-forming cells treated with OCT-saturated carriers and exposed to RMF at different frequencies in comparison to RMF-unexposed settings.

The results are presented as mean ± SEM calculated from the results obtained for all agar discs (C, M, B).

**Figure S6.** Percent of octenidine dihydrochloride extracted from all agar discs (C, M, B) exposed to RMF of 5 Hz and 50 Hz, in comparison to its initial concentration in the paper disc.

The results are presented as mean ± SEM calculated from the results obtained for all agar discs (C, M, B).

| **a** |
| --- |
| 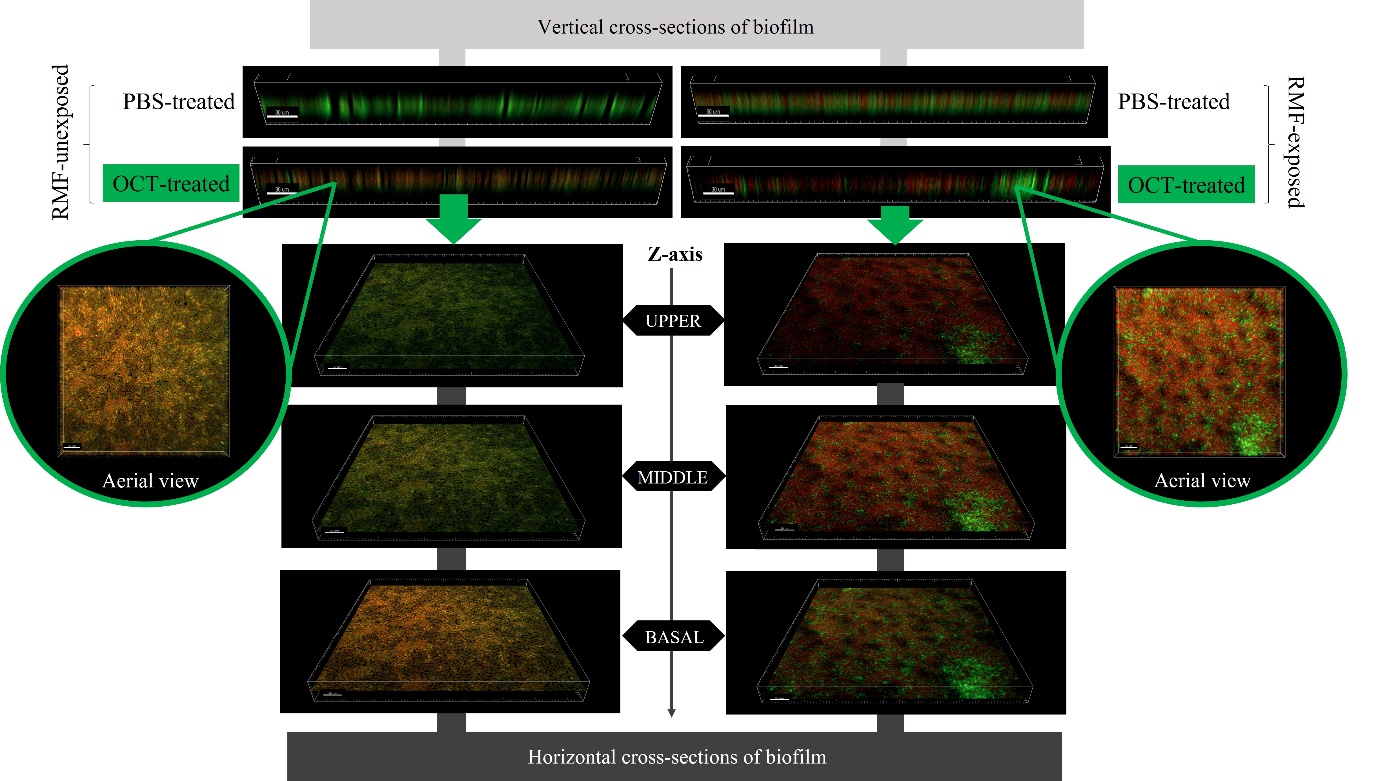 |
| **b** |
| 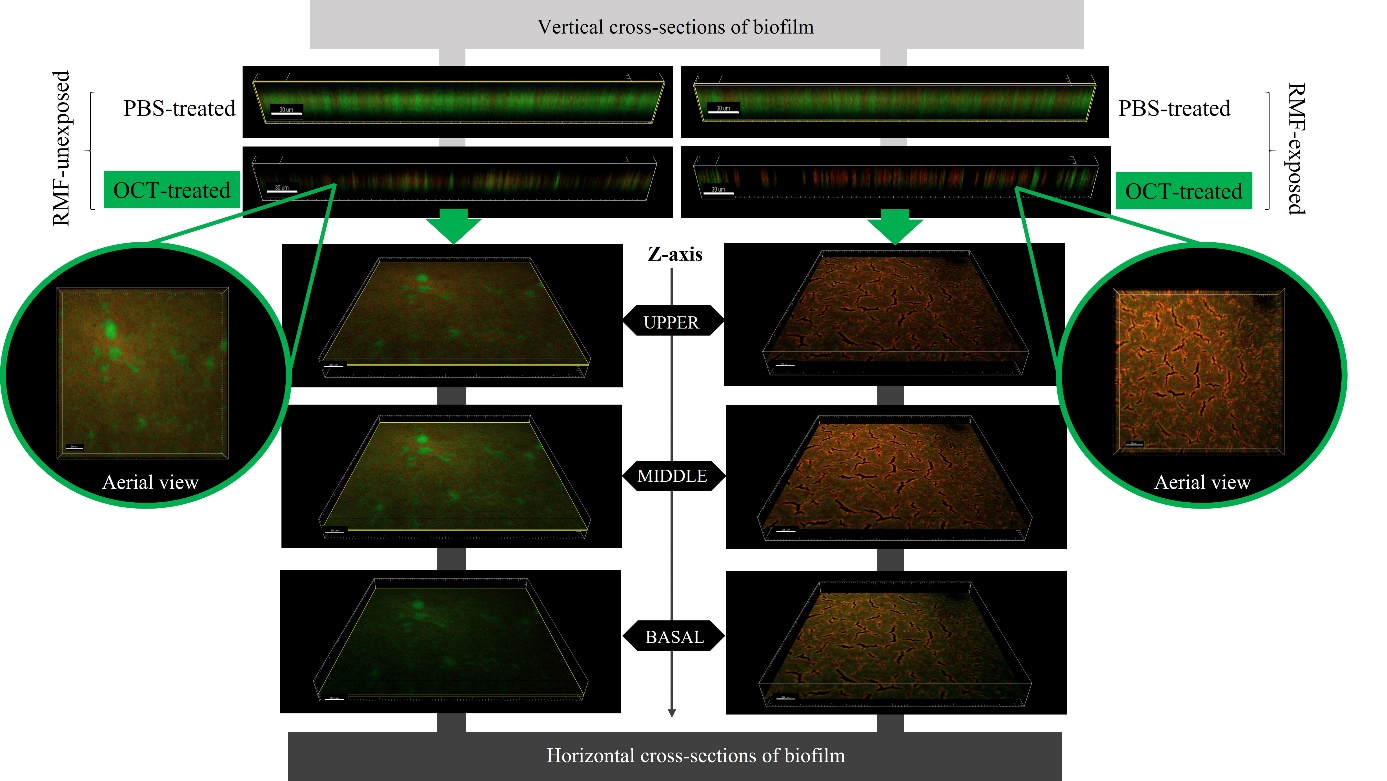 |

**Figure S7.** The spatial location of (**a**) staphylococcal and (**b**) pseudomonal cells within biofilm treated with OCT-saturated carriers and/or exposed to RMF (50 Hz).

The cells with non-altered cell walls dye green (as a result of SYTO-9), while the cells with compromised cell walls dye red/orange (as a result of propidium-iodide incorporation).

| **a** | **b** |
| --- | --- |
|  |  |
| **c** | |
| 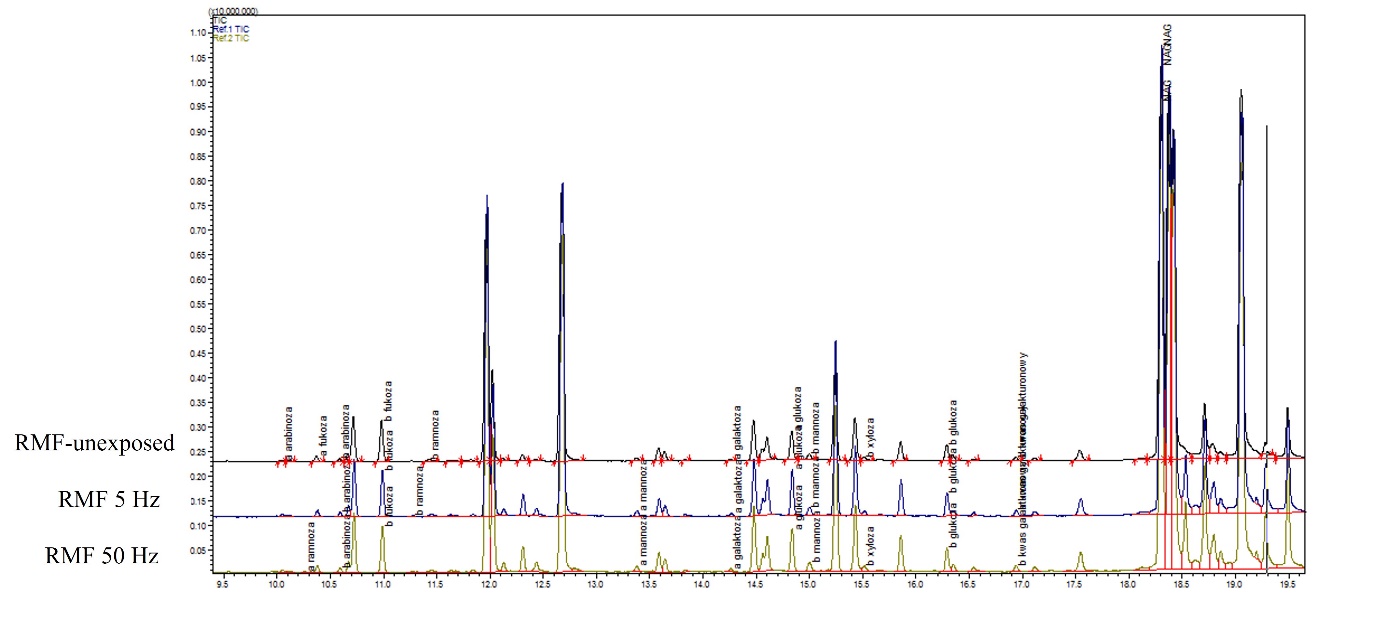 | |
| **d** | |
| 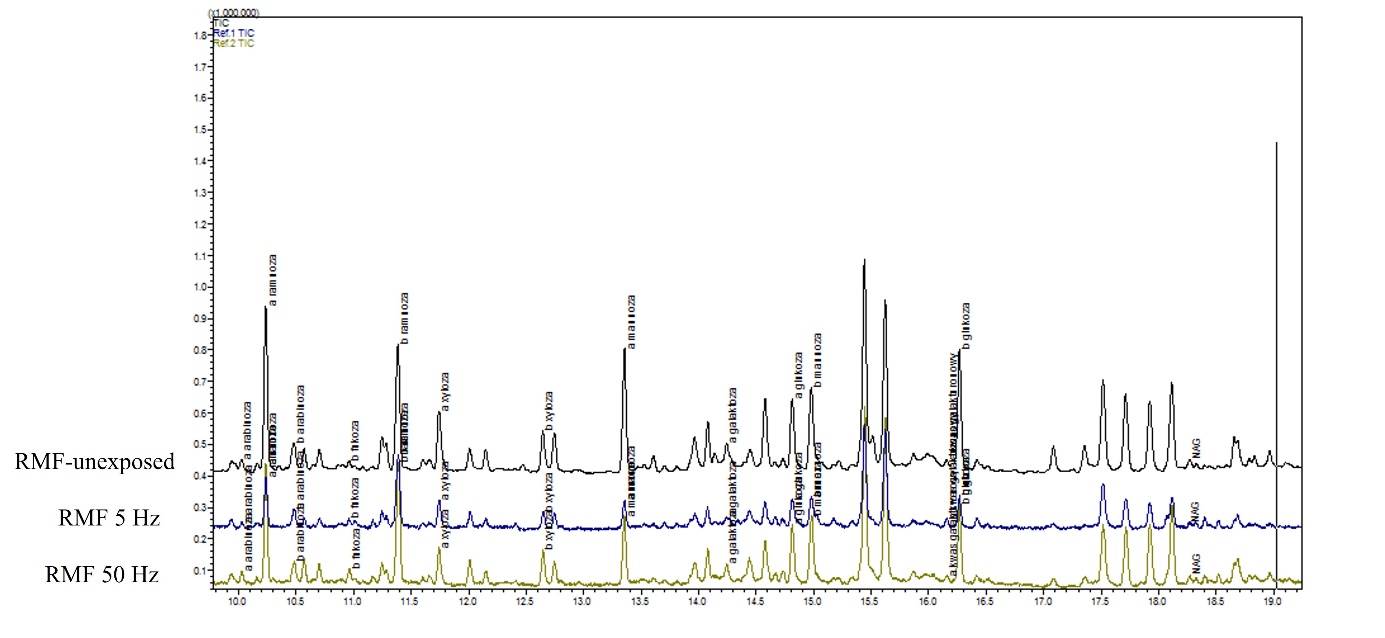 | |

**Figure S8.** The cell-free (**a**) pseudomonal and (**b**) staphylococcal biofilm matrix subjected to GC-MS/MS analysis (**c**, **d**, respectively). Unexposed - matrix obtained from RMF-unexposed biofilm; 5 Hz - matrix obtained from biofilm exposed to RMF of 5 Hz; 50 Hz - matrix obtained from biofilm exposed to RMF of 50 Hz.

**Table S2.** The values of magnetic induction inside the RMF generator, at the location of the biofilm samples depending on the applied AC frequency.

| **Magnetic induction [mT]** | **5 Hz** | **50 Hz** |
| --- | --- | --- |
| MIN | 8.352 | 8.698 |
| MAX | 8.354 | 8.700 |
| Average | 8.353 | 8.699 |

| **a** | **b** |
| --- | --- |
|  | 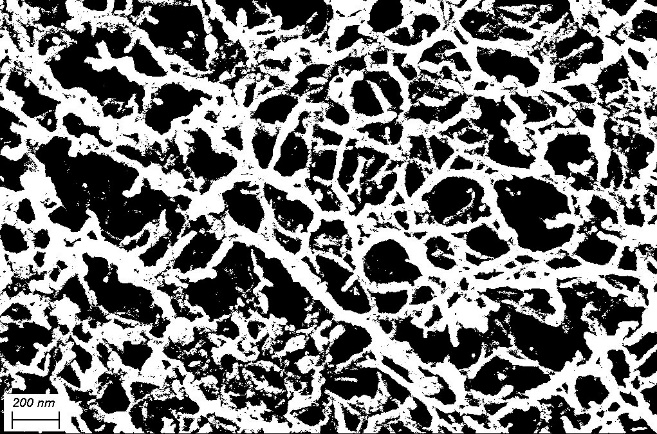 |

**Figure S9.** SEM-visualized structure of agar disk (**a**) and parametric processing of the image to assess agar porosity (**b**).

**The estimation of** **energy flux density of the electric field**

The relationship between the electric and magnetic field of an electromagnetic wave is presented by the system of Maxwell equations:

$$\varepsilon\varepsilon_{0}div\vec{E}= \rho$$

$$rot\vec{E}= - \mu\mu_{0}\frac{\partial\vec{H}}{\partial_{t}}$$

$div\vec{B}=0$ (1)

$$rot\vec{H}= \varepsilon\varepsilon_{0}\frac{\partial\vec{E}}{\partial_{t}}+ \vec{j}$$

where: $\vec{E}$ - vector of electric field intensity, $\vec{H}, \vec{B}$- vectors of magnetic field intensity and induction, respectively, $\varepsilon, \varepsilon_{0}$ - electric permeability of the medium and vacuum, $\mu,\mu_{0}$ - magnetic permeability of the medium and vacuum, $\rho$ - electric charge density, $\vec{j}$- electric current density.

Equation (1) shows, that the alternating magnetic field $(\frac{\partial\vec{H}}{\partial_{t}})$ creates a rotating electric field $(rot\vec{E})$. This equation expresses the content of Faraday's law of induction.

Maxwell's equations describing the propagation of an electromagnetic wave along the Z-axis have the following form:

$$\frac{\partial\vec{H}}{\partial_{z}}= -\varepsilon\varepsilon_{0}\frac{\partial E}{\partial_{t}}$$

$\frac{\partial E}{\partial_{z}}=-\mu\mu_{0}\frac{\partial H}{\partial_{t}}$ (2)

The above equations show that a change in one of the electromagnetic field vectors with time causes a change in the second vector in space.

The disturbance of the electromagnetic field propagating in space is related to energy, the density of which (energy contained in a unit of volume) is equal to the sum of the electric and magnetic field densities.

$u= \frac{1}{2}(\varepsilon\varepsilon_{0}E^{2}+ \mu\mu_{0}H^{2})$ (3)

After determining the partial derivatives in the Maxwell equations describing the electromagnetic wave (2), we get:

$\frac{1}{v}H= \varepsilon\varepsilon_{0}E$ (4)

where: $v= \frac{1}{\varepsilon\varepsilon_{0}\mu\mu_{0}}$- speed of propagation of an electromagnetic wave in a given medium.

So, substituting the velocity formula, we get:

$\sqrt{\mu\mu_{0}}H= \sqrt{\varepsilon\varepsilon_{0}}E$ (5)

Using the above dependence, the energy density of the electromagnetic field (3) can be represented as follows:

$u=\frac{1}{2} \left( \varepsilon\varepsilon_{0}E^{2}+ \mu\mu_{0}H^{2} \right)= \varepsilon\varepsilon_{0}E^{2}=\mu\mu_{0}H^{2}= \frac{1}{2\mu\mu_{0}}B^{2}$ (6)

Formula (6) directly shows that the energy density of the electric field is equal to the magnetic field density, and thus, given the magnetic induction values ​​and the magnetic and electric permeability coefficients of the medium and the vacuum, the value of the electric field can be calculated. In view of the above, an important quantity characterizing the interaction of the electric field on the surface perpendicular to the direction of propagation of the disturbance is the energy flux density, which can be expressed by the following relationship:

$J_{p}=E^{2}\frac{\sqrt{\varepsilon\varepsilon_{0}}}{\sqrt{\mu\mu_{0}}}$ (7)

The change in energy density in a charge-free region is related to the influence or outflow of the energy density flux equivalent to this change. When the disturbances spread in a medium containing free charges, the part of the energy that converts into Joule's heat should also be taken into account in the energy balance. In the RMF-generating system used in our study, the magnetic field lines are arranged orthonormally with respect to the biofilm-containing plates. In this case, the energy flux density affecting the sample caused by the generated electric field is negligible (the magnetic field was so large that only the observed and described effects related to magnetic fields were dominant).
